# Supplementary material for: Dual control of NAD+ synthesis by purine metabolites in yeast
Source: eLife. 2019 Mar 12;8:e43808. doi: 10.7554/eLife.43808 (PMC6430606; doi:10.7554/eLife.43808)
Supplement: Figure 2—source data 1. [file elife-43808-fig2-data1.pdf]

Figure 2, Figure 2_figure supplement 3 and Figure3_figure supplement 3

FY4 wild-type strain grown in SDcasaWU ± Adenine medium

Figure 2 and Figure 2- figure supplement 3

Peak area

	Metabolite (wavelength)	- Ade	- Ade	- Ade	- Ade	- Ade	- Ade	- Ade	- Ade	+ Ade	+ Ade	+ Ade	+ Ade	+ Ade	+ Ade	Mean - Ade	Mean + Ade	SD - Ade	SD + Ade	Unpaired T-test -Ade vs + Ade
Purines	Adenine (269 nm)	0.59	0.39	0.15	0.38	0.24	0.27	0.32		13.80	10.80	10.80	8.30	8.90	10.30	0.33	10.48	0.14	1.92	4.7E-05
	Adenosine (260 nm)	0.31	0.31	0.27	0.28	0.24	0.23		0.27	0.70	0.64	0.63	0.56	0.60		0.27	0.63	0.03	0.05	1.1E-05
	ADP (260 nm)	49.20	43.40	34.30	40.02	37.15	48.00	47.80		33.85	43.20	40.91	40.87	42.00	44.60	42.84	40.91	5.85	3.74	4.9E-01
	AMP (260 nm)	7.81	6.30	6.20	6.90	6.70	6.10	7.20	8.00	5.00	5.50	6.50		6.00	6.40	6.90	5.88	0.72	0.63	2.4E-02
	ATP (260 nm)	369.90	378.50	363.20	382.90	359.00	359.00	384.00	381.00	410.30	410.20	440.00	422.00	411.00	392.00	372.19	414.25	10.73	15.88	4.4E-04
	GDP (260 nm)	39.35	40.50	36.47	40.20	38.50	40.20	46.60	45.50	38.60	36.70	40.80	34.75	39.80	37.60	40.92	38.04	3.43	2.18	8.1E-02
	GTP (260 nm)	81.20	88.40	79.50	80.40	78.90	78.20	83.60	90.40	77.30	82.60	85.80	84.90	77.30	77.40	82.58	80.88	4.55	4.03	4.8E-01
	guanosine (260 nm)	0.32	0.25	0.13	0.23	0.14	0.14	0.30	0.24	0.74	0.73	0.84	0.86	1.02	1.08	0.22	0.88	0.07	0.14	1.9E-05
	Hypoxanthine (260 nm)	2.03	2.64	2.61	2.72	2.00	2.90			12.60	15.80	10.95	10.75	11.54	10.80	2.48	12.07	0.38	1.95	4.8E-05
	Inosine (254 nm)	1.07	0.64	0.52	1.10	0.47	0.48	0.59	0.40	4.75	4.72	5.17	4.42	4.20	4.54	0.66	4.63	0.27	0.33	7.0E-10
	SZMP (269 nm)	3.63	3.32	2.82	3.67	4.06	3.75	5.09	4.60	0.04	0.05	0.13	0.18	0.09	0.08	3.87	0.09	0.71	0.05	1.3E-06
	ZMP (269 nm)		1.95	1.75	1.53	1.69	1.75	2.52	2.85	0.18	0.18	0.23	0.27	0.18	0.26	2.01	0.22	0.49	0.04	6.4E-05
Pyrimidines	CDP (260 nm)	1.80	1.80	1.80	1.60	2.30	1.76	2.21	2.21		1.96	1.95	1.77	1.79	1.90	1.94	1.87	0.26	0.09	5.6E-01
	CMP (260 nm)	1.58	1.38	1.23	1.47	1.07	1.50	1.54	1.64	1.80	1.75	1.50	1.45	1.43	1.33	1.43	1.54	0.19	0.19	2.8E-01
	CTP (260 nm)	44.30	43.60	40.89	42.33	39.90	40.84	44.50	45.20	43.74	43.84	43.90		44.20	43.30	42.70	43.80	1.98	0.33	1.7E-01
	Cytidine (260 nm)	0.51	0.49	0.54	0.60	0.62	0.60	0.75	0.85	0.82	0.73	0.79	0.66	0.84	0.77	0.62	0.77	0.12	0.07	1.4E-02
	UDP (260 nm)	27.10	31.90	28.30	32.50	30.50	33.20	30.60		30.90	33.20	30.30		32.50	31.70	30.59	31.72	2.22	1.17	2.8E-01
	UDP-N-acetyl-glucosamine (260 nm)	31.80	29.80	29.60	30.20	26.70	27.80	29.20	32.00	34.70	32.20	31.60	32.60	29.20	29.50	29.64	31.63	1.80	2.06	8.7E-02
	Uracil (260 nm)	2.25	2.17	1.93	1.24	1.14	2.09	2.32	2.38	4.61	4.50	4.06	3.60	3.70	3.90	1.94	4.06	0.48	0.42	1.7E-06
	UTP (260 nm)	72.40	77.40	64.42	65.30	69.90	70.20	80.30	80.80	65.30	73.40	70.50		72.30	71.10	72.59	70.52	6.36	3.12	4.5E-01
Pyridines	3-hydroxy-Anthranilate (360 nm)	0.20	0.15	0.14	0.16	0.14	0.19	0.20	0.18	0.04	0.04	0.06	0.07	0.04	0.03	0.17	0.05	0.03	0.01	1.6E-07
	3-hydroxy-Kynurenine (390 nm)	0.36	0.36	0.30	0.34	0.30	0.31	0.27	0.26	0.11	0.10	0.07	0.06	0.07	0.06	0.31	0.08	0.04	0.02	7.7E-09
	Kynurenine (360 nm)	0.46	0.46	0.39	0.43	0.39	0.42	0.46	0.47	0.20	0.21	0.25	0.25	0.21	0.21	0.43	0.22	0.03	0.02	8.0E-09
	NAD^+ (260 nm)	9.20	10.45	11.05	10.85	10.60	10.48	10.42	9.97	11.09	11.30	11.60	11.10	11.97	11.27	10.38	11.39	0.57	0.34	1.5E-03
	NADH (340 nm)	33.90	36.05	33.90	34.30	38.30	33.24	36.54	35.90	38.70	40.80	30.99	30.96	30.60	35.60	35.27	34.61	1.72	4.44	7.4E-01
	NADPH (340 nm)	6.10	5.45	4.90	6.20	6.23	5.25	5.36	6.50	5.60	5.20	4.68	5.90	4.60	6.20	5.75	5.36	0.58	0.65	2.8E-01
	Nicotinic acid (260 nm)	2.82	2.73	1.98	2.38	2.07	2.38	2.84	2.89	1.70	1.88	1.44	1.42	1.76	1.99	2.51	1.70	0.36	0.23	2.6E-04
Aminoacids	Phenylalanine (260 nm)	1.76	1.76	1.43	1.77	1.46	1.69	1.80	1.70	1.73	1.75	2.28	2.08	1.64	1.68	1.67	1.86	0.14	0.26	1.5E-01
	Tryptophan (280 nm)	36.80	35.20	31.60	33.40	31.80	33.80	36.40	37.40	46.30	47.30	48.92	43.88	44.60	44.90	34.55	45.98	2.24	1.90	3.0E-07
	Tyrosine (295 nm)	8.57	8.17	8.25	8.44	8.40	8.80	8.46	8.70	9.86	9.50		9.30	8.40	9.30	8.47	9.27	0.21	0.54	2.6E-02
Other metabolites	Fumarate (260 nm)	1.85	1.50	1.78	1.82	1.48	1.52	1.48	1.63	0.58	0.44	0.48	0.47	0.44	0.47	1.63	0.48	0.16	0.05	1.8E-08
	Thiamine (340 nm)	5.90	5.79	4.37	5.11	4.16	4.94	5.75	5.79	4.77	5.03	4.87		4.72	4.96	5.23	4.87	0.69	0.13	1.9E-01
	Thyamine pyrophosphate (280 nm)	12.20	11.90	11.20	12.00	10.79	11.70	11.90	12.40	11.10	11.60	13.52	11.08	11.30	11.04	11.76	11.61	0.53	0.96	7.3E-01

Non-determinable for technical reasons
mostly due to co-elution
in some samples

p>0.05
0.05<p>0.01
0.01<p>0.001
p<0.001

Figure 2 and Figure 2- figure supplement 3

Relative peak area **(mean peak area from cells grown in the presence of adenine was set at 1 and used to calculate the relative peak areas)**

	Metabolite	- Ade	- Ade	- Ade	- Ade	- Ade	- Ade	- Ade	- Ade	+ Ade	+ Ade	+ Ade	+ Ade	+ Ade	+ Ade	Mean - Ade	Mean + Ade	SD - Ade	SD + Ade	Unpaired T-test -Ade vs + Ade
Purines	Adenine	0.06	0.04	0.01	0.04	0.02	0.03	0.03		1.32	1.03	1.03	0.79	0.85	0.98	0.03	1.00	0.01	0.18	4.7E-05
	Adenosine	0.50	0.49	0.43	0.45	0.38	0.37		0.43	1.12	1.02	1.00	0.89	0.96		0.44	1.00	0.05	0.08	1.1E-05
	ADP	1.20	1.06	0.84	0.98	0.91	1.17	1.17		0.83	1.06	1.00	1.00	1.03	1.09	1.05	1.00	0.14	0.09	4.9E-01
	AMP	1.33	1.07	1.05	1.17	1.14	1.04	1.22	1.36	0.85	0.94	1.11		1.02	1.09	1.17	1.00	0.12	0.11	2.4E-02
	ATP	0.89	0.91	0.88	0.92	0.87	0.87	0.93	0.92	0.99	0.99	1.06	1.02	0.99	0.95	0.90	1.00	0.03	0.04	4.4E-04
	GDP	1.03	1.06	0.96	1.06	1.01	1.06	1.22	1.20	1.01	0.96	1.07	0.91	1.05	0.99	1.08	1.00	0.09	0.06	8.1E-02
	GTP	1.00	1.09	0.98	0.99	0.98	0.97	1.03	1.12	0.96	1.02	1.06	1.05	0.96	0.96	1.02	1.00	0.06	0.05	4.8E-01
	guanosine	0.36	0.28	0.14	0.27	0.16	0.16	0.34	0.27	0.85	0.83	0.96	0.98	1.16	1.23	0.25	1.00	0.08	0.16	1.9E-05
	Hypoxanthine	0.17	0.22	0.22	0.23	0.17	0.24			1.04	1.31	0.91	0.89	0.96	0.89	0.21	1.00	0.03	0.16	4.8E-05
	Inosine	0.23	0.14	0.11	0.24	0.10	0.10	0.13	0.09	1.03	1.02	1.12	0.95	0.91	0.98	0.14	1.00	0.06	0.07	7.0E-10
	SZMP	39.17	35.83	30.43	39.60	43.81	40.47	54.93	49.64	0.43	0.55	1.35	1.89	0.93	0.85	41.74	1.00	7.71	0.54	1.3E-06

	ZMP		9.03	8.10	7.08	7.82	8.10	11.67	13.19	0.85	0.81	1.06	1.25	0.81	1.20	9.29	1.00	2.27	0.20	6.4E-05
Pyrimidines	CDP	0.96	0.96	0.96	0.85	1.23	0.94	1.18	1.18		1.05	1.04	0.94	0.96	1.01	1.03	1.00	0.14	0.05	5.6E-01
	CMP	1.02	0.89	0.80	0.95	0.69	0.97	1.00	1.06	1.17	1.13	0.97	0.94	0.93	0.86	0.92	1.00	0.12	0.12	2.8E-01
	CTP	1.01	1.00	0.93	0.97	0.91	0.93	1.02	1.03	1.00	1.00	1.00		1.01	0.99	0.97	1.00	0.05	0.01	1.7E-01
	Cytidine	0.67	0.64	0.70	0.78	0.81	0.78	0.98	1.11	1.07	0.95	1.03	0.86	1.09	1.00	0.81	1.00	0.16	0.09	1.4E-02
	UDP	0.85	1.01	0.89	1.02	0.96	1.05	0.96		0.97	1.05	0.96		1.02	1.00	0.96	1.00	0.07	0.04	2.8E-01
	UDP-N-acetyl-glucosamine	1.01	0.94	0.94	0.95	0.84	0.88	0.92	1.01	1.10	1.02	1.00	1.03	0.92	0.93	0.94	1.00	0.06	0.06	8.7E-02
	Uracil	0.55	0.53	0.48	0.31	0.28	0.51	0.57	0.59	1.14	1.11	1.00	0.89	0.91	0.96	0.48	1.00	0.12	0.10	1.7E-06
	UTP	1.03	1.10	0.91	0.93	0.99	1.00	1.14	1.15	0.93	1.04	1.00		1.03	1.01	1.03	1.00	0.09	0.04	4.5E-01
Pyridines	3-hydroxy-Anthranilate	4.36	3.20	2.95	3.46	3.03	4.04	4.19	3.93	0.79	0.88	1.28	1.45	0.90	0.70	3.64	1.00	0.55	0.30	1.6E-07
	3-hydroxy-Kynurenine	4.54	4.54	3.81	4.29	3.79	3.91	3.48	3.29	1.39	1.27	0.88	0.81	0.87	0.78	3.96	1.00	0.47	0.26	7.7E-09
	Kynurenine	2.08	2.08	1.75	1.94	1.74	1.91	2.08	2.12	0.90	0.95	1.14	1.13	0.96	0.93	1.96	1.00	0.15	0.10	8.0E-09
	NAD+	0.81	0.92	0.97	0.95	0.93	0.92	0.91	0.88	0.97	0.99	1.02	0.97	1.05	0.99	0.91	1.00	0.05	0.03	1.5E-03
	NADH	0.98	1.04	0.98	0.99	1.11	0.96	1.06	1.04	1.12	1.18	0.90	0.89	0.88	1.03	1.02	1.00	0.05	0.13	7.4E-01
	NADPH	1.14	1.02	0.91	1.16	1.16	0.98	1.00	1.21	1.04	0.97	0.87	1.10	0.86	1.16	1.07	1.00	0.11	0.12	2.8E-01
	Nicotinic acid	1.66	1.61	1.17	1.40	1.22	1.40	1.67	1.70	1.00	1.11	0.85	0.84	1.04	1.17	1.48	1.00	0.21	0.14	2.6E-04
Aminoacids	Phenylalanine	0.95	0.95	0.77	0.95	0.78	0.91	0.97	0.91	0.93	0.94	1.23	1.12	0.88	0.90	0.90	1.00	0.08	0.14	1.5E-01
	Tryptophan	0.80	0.77	0.69	0.73	0.69	0.74	0.79	0.81	1.01	1.03	1.06	0.95	0.97	0.98	0.75	1.00	0.05	0.04	3.0E-07
	Tyrosine	0.92	0.88	0.89	0.91	0.91	0.95	0.91	0.94	1.06	1.02		1.00	0.91	1.00	0.91	1.00	0.02	0.06	2.6E-02
Other metabolites	Fumarate	3.85	3.13	3.71	3.79	3.08	3.17	3.08	3.40	1.21	0.92	1.00	0.98	0.92	0.98	3.40	1.00	0.33	0.11	1.8E-08
	Thiamine	1.21	1.19	0.90	1.05	0.85	1.01	1.18	1.19	0.98	1.03	1.00		0.97	1.02	1.07	1.00	0.14	0.03	1.9E-01
	Thyamine pyrophosphate	1.05	1.03	0.96	1.03	0.93	1.01	1.03	1.07	0.96	1.00	1.16	0.95	0.97	0.95	1.01	1.00	0.05	0.08	7.3E-01

Non-determinable for technical reasons
mostly due to co-elution
in some samples

p>0.05
0.05<p>0.01
0.01<p>0.001
p<0.001

Figure 3_figure supplement 3

Content
(nmol)

															Mean	Mean	SD	SD	Unpaired
	- Ade	- Ade	- Ade	- Ade	- Ade	- Ade	- Ade	- Ade	+ Ade	+ Ade	+ Ade	+ Ade	+ Ade	+ Ade	- Ade	+ Ade	- Ade	+ Ade	T-test -Ade vs + Ade
ATP	19.27	19.71	18.92	19.94	18.70	18.70	20.00	19.84	21.37	21.36	22.92	21.98	21.41	20.42	0.04	0.05	0.00	0.00	4.4E-04
ADP	2.93	2.58	2.04	2.38	2.21	2.86	2.85		2.01	2.57	2.44	2.43	2.50	2.65	0.00	0.00	0.00	0.00	4.9E-01
AMP	0.40	0.32	0.32	0.36	0.35	0.31	0.37	0.41	0.26	0.28	0.34		0.31	0.33	0.18	0.05	0.02	0.01	2.4E-02
AXP	22.60	22.62	21.28	22.68	21.25	21.87	23.22		23.64	24.22	25.69		24.22	23.40	22.22	24.23	0.76	0.89	3.5E-03
AEC	0.91738	0.92855	0.937	0.9318	0.93173	0.9203	0.92274		0.94649	0.93521	0.93956		0.93561	0.92918	0.93	0.94	0.01	0.01	2.9E-02

Relative content (mean AXP content from cells grown in the presence of adenine was set at 1 and used to calculate relative content)

															Mean	Mean	SD	SD	Unpaired
	- Ade	- Ade	- Ade	- Ade	- Ade	- Ade	- Ade	- Ade	+ Ade	+ Ade	+ Ade	+ Ade	+ Ade	+ Ade	- Ade	+ Ade	- Ade	+ Ade	T-test -Ade vs + Ade
AXP	0.93	0.93	0.88	0.94	0.88	0.90	0.96		0.98	1.00	1.06		1.00	0.97	0.92	1.00	0.03	0.04	3.5E-03

nmol/peak area unit (determined with pure compounds as described in the Material and Methods section

ATP	19.2
ADP	16.8
AMP	19.4

p>0.05
0.05<p>0.01
0.01<p>0.001
p<0.001

Non-determinable for technical reasons
mostly due to co-elution
in some samples
