# Supplementary material for: Dual control of NAD+ synthesis by purine metabolites in yeast
Source: eLife. 2019 Mar 12;8:e43808. doi: 10.7554/eLife.43808 (PMC6430606; doi:10.7554/eLife.43808)
Supplement: Figure 2—figure supplement 2—source data 1. [file elife-43808-fig2-figsupp2-data1.pdf]

Figure 2_figure supplement 2B

FY4 prototrophic cells grown in SDcasaWU medium ± Adenine

																	Mean	Mean	SD	SD	Unpaired t-test
	- Ade	- Ade	- Ade	- Ade	- Ade	- Ade	- Ade	- Ade	+ Ade	+ Ade	+ Ade	+ Ade	+ Ade	+ Ade	+ Ade	+ Ade	- Ade	+ Ade	- Ade	+ Ade	- Ade vs + Ade
**Median cell volume**	44.9	49.3	50.1	47.8	47.7	49.5	49.4	49.1	55.3	55.8	53.9	54	55.4	54.7	55.5	55.9	51.13	54.90	3.49	0.83	1.5E-06

p>0.05
0.05<p>0.01
0.01<p>0.001
p<0.001
