# Supplementary material for: Dual control of NAD+ synthesis by purine metabolites in yeast
Source: eLife. 2019 Mar 12;8:e43808. doi: 10.7554/eLife.43808 (PMC6430606; doi:10.7554/eLife.43808)
Supplement: Figure 2—figure supplement 5—source data 1. [file elife-43808-fig2-figsupp5-data1.pdf]

Figure 2 _ figure supplement 5

Different wild-type strains grown in SDcasaWU ± Adenine medium

Peak area

Metabolite/Strain	- Ade	- Ade	- Ade	- Ade	- Ade	- Ade	- Ade	- Ade	- Ade	+ Ade	+ Ade	+ Ade	+ Ade	+ Ade	+ Ade	+ Ade	+ Ade	Mean - Ade	Mean + Ade	SD - Ade	SD + Ade	Unpaired t-Test - Ade vs + Ade
ATP/WT1	363.2	382.9	369.9	378.5	363.2	382.9	359	359	384	410.3	410.2	411	392	410.3	410.2	440	422	371.40	413.25	10.71	13.55	8.1E-06
ATP/WT2	353.2	359.11	366.4	381.5	364.5	352.4				417.96	452	416.4	415.6	422	430.2			362.85	425.69	10.77	13.96	8.2E-06
ATP/WT3	401.9	322.6	376.8	353.5	358.3	364.2				449.4	454.2	423.7	435.54	428.8	424			362.88	435.94	26.27	13.10	4.1E-04
NAD+/WT1	9.2	10.45	11.39	11.15	10.6	10.43	10.62	10.15	9.97	11.09	11.3	11.6	11.1	11.09	11.27	11.62	11.97	10.44	11.38	0.64	0.32	2.2E-03
NAD+/WT2	15.58	22.43	18.87	23.63	20.4	22.1				23.95	25.66	25.57	25.8	25.6	28.7			20.50	25.88	2.93	1.54	4.6E-03
NAD+/WT3	20.77	21.12	22.1	22.5	22.81	23.7				23.42	27.2	24.6	26.5	28.72	28.57			22.17	26.50	1.09	2.14	2.6E-03

Relative peak area (mean peak area from cells grown in the presence of adenine was set at 1 and used to calculate the relative peak areas)

Metabolite/Strain	- Ade	- Ade	- Ade	- Ade	- Ade	- Ade	- Ade	- Ade	- Ade	+ Ade	+ Ade	+ Ade	+ Ade	+ Ade	+ Ade	+ Ade	+ Ade	Mean - Ade	Mean + Ade	SD - Ade	SD + Ade	Unpaired t-Test - Ade vs + Ade
ATP/WT1	0.88	0.93	0.90	0.92	0.88	0.93	0.87	0.87	0.93	0.99	0.99	0.99	0.95	0.99	0.99	1.06	1.02	0.90	1.00	0.03	0.03	8.1E-06
ATP/WT2	0.83	0.84	0.86	0.90	0.86	0.83				0.98	1.06	0.98	0.98	0.99	1.01			0.85	1.00	0.03	0.03	8.2E-06
ATP/WT3	0.92	0.74	0.86	0.81	0.82	0.84				1.03	1.04	0.97	1.00	0.98	0.97			0.83	1.00	0.06	0.03	4.1E-04
NAD+/WT1	0.81	0.92	1.00	0.98	0.93	0.92	0.93	0.89	0.88	0.97	0.99	1.02	0.98	0.97	0.99	1.02	1.05	0.92	1.00	0.06	0.03	2.2E-03
NAD+/WT2	0.60	0.87	0.73	0.91	0.79	0.85				0.93	0.99	0.99	1.00	0.99	1.11			0.79	1.00	0.11	0.06	4.6E-03
NAD+/WT3	0.78	0.80	0.83	0.85	0.86	0.89				0.88	1.03	0.93	1.00	1.08	1.08			0.84	1.00	0.04	0.08	2.6E-03

WT1 : FY4
WT2: Y286
WT3: BY4742

p>0.05
0.05<p>0.01
0.01<p>0.001
p<0.001
