# Supplementary material for: Dual control of NAD+ synthesis by purine metabolites in yeast
Source: eLife. 2019 Mar 12;8:e43808. doi: 10.7554/eLife.43808 (PMC6430606; doi:10.7554/eLife.43808)
Supplement: Figure 3—source data 1. [file elife-43808-fig3-data1.pdf]

Figure 3 A-B

Wild-type and mutant strains grown in SDcasaWU ± Adenine medium

Figure 3 A-B

Peak area

Metabolite/Strain	- Ade	- Ade	- Ade	- Ade	- Ade	- Ade	+ Ade	+ Ade	+ Ade	+ Ade	+ Ade	+ Ade	Mean - Ade	Mean + Ade	SD - Ade	SD + Ade	Unpaired t-Test -Ade vs + Ade	Unpaired t-Test mutant - Ade *vs* WT - Ade	Unpaired t-Test mutant + Ade *vs* WT + Ade
ATP/WT1	413	399	377	401	382	401	467	465	466	480	456	452	395.50	464.33	13.44	9.77	2.8E-06		
ATP/*adk1*	176	145	181	188	167	150	204	205	255	261	259	278	167.83	243.67	17.24	31.34	9.1E-04	5.0E-10	3.4E-06
ATP/*kcs1*	867	948	930	887	890	932	1025	1026	1031	1003	931		909.00	1003.20	31.94	41.78	3.8E-03	5.8E-09	4.2E-06
NAD+/WT	18.27		21.08	17.16	21.9	21.3		26.1	27.4	29.9	24.3	23.9	19.94	26.32	2.09	2.45	2.3E-03		
NAD+/*adk1*	6.84	5.23	5.3	7.1	7.1	6.8	8.3	7.03	7.69	7.9	8.05	8.5	6.40	7.91	0.88	0.52	6.6E-03	3.1E-05	4.7E-05
NAD+/*kcs1*	25.4		25.7	27.07	24.5		30.1		33.83	33.2	30.3		25.67	31.86	1.07	1.93	3.1E-03	1.7E-03	6.8E-03

Non-determinable for technical reasons
mostly due to co-elution
in some samples

Relative peak area **(mean peak area from cells grown in the presence of adenine was set at 1 and used to calculate the relative peak areas)**

Figure 3 A-B

Metabolite/Strain	- Ade	- Ade	- Ade	- Ade	- Ade	- Ade	+ Ade	+ Ade	+ Ade	+ Ade	+ Ade	+ Ade	Mean - Ade	Mean + Ade	SD - Ade	SD + Ade	Unpaired t-Test - Ade vs + Ade	Unpaired t-Test mutant - Ade *vs* WT - Ade	Unpaired t-Test mutant + Ade *vs* WT + Ade
ATP/WT1	0.89	0.86	0.81	0.86	0.82	0.86	1.01	1.00	1.00	1.03	0.98	0.97	0.85	1.00	0.03	0.02	2.8E-06		
ATP/*adk1*	0.38	0.31	0.39	0.40	0.36	0.32	0.44	0.44	0.55	0.56	0.56	0.60	0.36	0.52	0.04	0.07	9.1E-04	5.0E-10	3.4E-06
ATP/*kcs1*	1.87	2.04	2.00	1.91	1.92	2.01	2.21	2.21	2.22	2.16	2.01		1.96	2.16	0.07	0.09	3.8E-03	5.8E-09	4.2E-06
NAD+/WT	0.69		0.80	0.65	0.83	0.81		0.99	1.04	1.14	0.92	0.91	0.76	1.00	0.08	0.09	2.3E-03		
NAD+/*adk1*	0.26	0.20	0.20	0.27	0.27	0.26	0.32	0.27	0.29	0.30	0.31	0.32	0.24	0.30	0.03	0.02	6.6E-03	3.1E-05	4.7E-05
NAD+/*kcs1*	0.97		0.98	1.03	0.93		1.14		1.29	1.26	1.15		0.98	1.21	0.04	0.07	3.1E-03	1.7E-03	6.8E-03

Non-determinable for technical reasons
mostly due to co-elution
in some samples

p>0.05
0.05<p>0.01
0.01<p>0.001
p<0.001
