# Supplementary material for: Dual control of NAD+ synthesis by purine metabolites in yeast
Source: eLife. 2019 Mar 12;8:e43808. doi: 10.7554/eLife.43808 (PMC6430606; doi:10.7554/eLife.43808)
Supplement: Figure 3—source data 2. [file elife-43808-fig3-data2.pdf]

Figure 3 C-D

Wild-type and mutant strains grown in SDcasaWU + Adenine ± nicotinic acid medium

Peak area

Metabolite/Strain	- NA	- NA	- NA	- NA	- NA	- NA	+ NA	+ NA	+ NA	+ NA	+ NA	+ NA	Mean - NA	Mean + NA	SD - NA	SD + NA	Unpaired t-Test - Ade vs + Ade
ATP/WT	387	337	381	373			390.3	383	387.9	387.43			369.50	387.16	22.41	3.04	2.1E-01
ATP/*adk1*	153	163	152	161			138.5	150	139	118			157.25	136.38	5.56	13.35	4.5E-02
ATP/*kcs1*	664.6	673.6	656.2	666.8	644.6	664.2	650.6	602.2	596	578	617.2	578	661.67	603.67	10.05	27.44	2.4E-03
NAD+/WT	10.98	7.2	7.55	8.34			22.1	21.71	21.2	21.75			8.52	21.69	1.71	0.37	3.8E-04
NAD+/*adk1*	9.2	7.1	6.2	6.9			10.8	7.03	6.8	6.9			7.35	7.88	1.29	1.95	6.7E-01
NAD+/*kcs1*	13.2	13.8	12	16.9	12.6	12	34.2	31	28.8	30.8	29.6	27.8	13.42	30.37	1.84	2.23	7.9E-08

Relative peak area **(mean peak area from cells grown in the presence of nicotinic acid was set at 1 and used to calculate the relative peak areas)**

Metabolite/Strain	- NA	- NA	- NA	- NA	- NA	- NA	+ NA	+ NA	+ NA	+ NA	+ NA	+ NA	Mean - NA	Mean + NA	SD - NA	SD + NA	Unpaired t-Test - Ade vs + Ade
ATP/WT	1.00	0.87	0.98	0.96			1.01	0.99	1.00	1.00			0.95	1.00	0.06	0.01	2.1E-01
ATP/*adk1*	0.40	0.42	0.39	0.42			0.36	0.39	0.36	0.30			0.41	0.35	0.01	0.03	4.5E-02
ATP/*kcs1*	1.72	1.74	1.69	1.72	1.66	1.72	1.68	1.56	1.54	1.49	1.59	1.49	1.71	1.56	0.03	0.07	2.4E-03
NAD+/WT	0.51	0.33	0.35	0.38			1.02	1.00	0.98	1.00			0.39	1.00	0.08	0.02	3.8E-04
NAD+/*adk1*	0.42	0.33	0.29	0.32			0.50	0.32	0.31	0.32			0.34	0.36	0.06	0.09	6.7E-01
NAD+/*kcs1*	0.61	0.64	0.55	0.78	0.58	0.55	1.58	1.43	1.33	1.42	1.36	1.28	0.62	1.40	0.09	0.10	7.9E-08

-NA: - Nicotinic Acid
+ NA: + Nicotinic Ac.

p>0.05
0.05<p>0.01
0.01<p>0.001
p<0.001
