# Supplementary material for: Dual control of NAD+ synthesis by purine metabolites in yeast
Source: eLife. 2019 Mar 12;8:e43808. doi: 10.7554/eLife.43808 (PMC6430606; doi:10.7554/eLife.43808)
Supplement: Figure 3—figure supplement 1—source data 1. [file elife-43808-fig3-figsupp1-data1.pdf]

Figure 3_figure supplement 1

Wild-type and mutant strains grown in SDcasaWU ± Adenine medium

Peak area

													Mean	Mean	SD	SD	Unpaired t-Test	Unpaired t-Test	Unpaired t-Test
**Metabolite/Strain**	**- Ade**	**- Ade**	**- Ade**	**- Ade**	**- Ade**	**- Ade**	**+ Ade**	**+ Ade**	**+ Ade**	**+ Ade**	**+ Ade**	**+ Ade**	**- Ade**	**+ Ade**	**- Ade**	**+ Ade**	**-Ade vs + Ade**	**mutant - Ade *vs* WT - Ade**	**mutant + Ade *vs* WT + Ade**
**NADH/WT**	32.6	34.3	30.7	37.5	33.4	32.2	30.7	34.6	33.2	37.5	35.7	35.2	33.45	34.48	2.32	2.33	4.6E-01		
**NADH/*adk1***	17.2	15.1	15.6	15	14.8	16.5	17.3	14.8	19.3	14.3	16.4		15.70	16.42	0.95	2.01	4.9E-01	8.9E-07	2.4E-07
**NADH/*kcs1***	39.4	53.6		48.7	43.3	53.6	40.5	40.8	42.3	49.6	48.3	45.6	47.72	44.52	6.30	3.90	3.6E-01	5.1E-03	6.0E-04

Non-determinable for technical reasons mostly due to co-elution in some samples

Relative peak area (mean peak area from cells grown in the presence of adenine was set at 1 and used to calculate the relative peak areas)

													Mean	Mean	SD	SD	Unpaired t-Test	Unpaired t-Test	Unpaired t-Test
**Metabolite/Strain**	**- Ade**	**- Ade**	**- Ade**	**- Ade**	**- Ade**	**- Ade**	**+ Ade**	**+ Ade**	**+ Ade**	**+ Ade**	**+ Ade**	**+ Ade**	**- Ade**	**+ Ade**	**- Ade**	**+ Ade**	**-Ade vs + Ade**	**mutant - Ade vs WT - Ade**	**mutant + Ade vs WT + Ade**
**NADH/WT**	0.9454	0.9947	0.8903	1.0875	0.9686	0.9338	0.8903	1.0034	0.9628	1.0875	1.0353	1.0208	0.97	1.00	0.07	0.07	4.6E-01		
**NADH/adk1**	0.4988	0.4379	0.4524	0.435	0.4292	0.4785	0.5017	0.4292	0.5597	0.4147	0.4756		0.46	0.48	0.03	0.06	4.9E-01	8.9E-07	2.4E-07
**NADH/kcs1**	1.1426	1.5544		1.4123	1.2557	1.5544	1.1745	1.1832	1.2267	1.4384	1.4007	1.3224	1.38	1.29	0.18	0.11	3.6E-01	5.1E-03	6.0E-04

Non-determinable for technical reasons mostly due to co-elution in some samples

$p>0.05$
$0.05<p>0.01$
$0.01<p>0.001$
$p<0.001$
