# Supplementary material for: Dual control of NAD+ synthesis by purine metabolites in yeast
Source: eLife. 2019 Mar 12;8:e43808. doi: 10.7554/eLife.43808 (PMC6430606; doi:10.7554/eLife.43808)
Supplement: Figure 3—figure supplement 2—source data 1. [file elife-43808-fig3-figsupp2-data1.pdf]

Figure 3- figure supplement 2 A-B

WT and *prs3* knock-out strains grown in SDcasaWU ± Adenine medium

Peak area

									Mean	Mean	SD	SD	Unpaired t-Test	Unpaired t-Test	Unpaired t-Test
**Metabolite/Strain**	**- Ade**	**- Ade**	**- Ade**	**- Ade**	**+ Ade**	**+ Ade**	**+ Ade**	**+ Ade**	**- Ade**	**+ Ade**	**- Ade**	**+ Ade**	**- Ade vs + Ade**	**mutant - Ade vs WT - Ade**	**mutant + Ade vs WT + Ade**
**ATP/WT**	163.1	160.2	163.9	164.6	178.7	177.5	176.4	178	162.95	177.65	1.93	0.97	9.0E-05		
**ATP/*prs3***	48.2	52.05	68.4	69.8	97.3	98.1	120.6	110.6	59.61	106.65	11.08	11.12	9.7E-04	2.4E-04	9.7E-04

									Mean	Mean	SD	SD	Unpaired t-Test	Unpaired t-Test	Unpaired t-Test
**Metabolite/Strain**	**- Ade**	**- Ade**	**- Ade**	**- Ade**	**+ Ade**	**+ Ade**	**+ Ade**	**+ Ade**	**- Ade**	**+ Ade**	**- Ade**	**+ Ade**	**- Ade vs + Ade**	**mutant - Ade vs WT - Ade**	**mutant + Ade vs WT + Ade**
**NAD^+/WT**	4.82	5.1	5.7	5.7	6.1	6.3	7	6.6	5.33	6.50	0.44	0.39	7.7E-03		
**NAD^+/*prs3***	1.92	2.5	2.8	2.85	3.99	4.3	4.2	4.7	2.52	4.30	0.43	0.30	7.7E-04	9.7E-05	1.6E-04

Relative peak area **(mean peak area from cells grown in the presence of adenine was set at 1 and used to calculate the relative peak areas)**

									Mean	Mean	SD	SD	Unpaired t-Test	Unpaired t-Test	Unpaired t-Test
**Metabolite/Strain**	**- Ade**	**- Ade**	**- Ade**	**- Ade**	**+ Ade**	**+ Ade**	**+ Ade**	**+ Ade**	**- Ade**	**+ Ade**	**- Ade**	**+ Ade**	**- Ade vs + Ade**	**mutant - Ade vs WT - Ade**	**mutant + Ade vs WT + Ade**
**ATP/WT**	0.92	0.90	0.92	0.93	1.01	1.00	0.99	1.00	0.92	1.00	0.01	0.01	9.0E-05		
**ATP/*prs3***	0.27	0.29	0.39	0.39	0.55	0.55	0.68	0.62	0.34	0.60	0.06	0.06	9.7E-04	2.4E-04	9.7E-04

									Mean	Mean	SD	SD	Unpaired t-Test	Unpaired t-Test	Unpaired t-Test
**Metabolite/Strain**	**- Ade**	**- Ade**	**- Ade**	**- Ade**	**+ Ade**	**+ Ade**	**+ Ade**	**+ Ade**	**- Ade**	**+ Ade**	**- Ade**	**+ Ade**	**- Ade vs + Ade**	**mutant - Ade vs WT - Ade**	**mutant + Ade vs WT + Ade**
**NAD^+/WT**	0.74	0.78	0.88	0.88	0.94	0.97	1.08	1.02	0.82	1.00	0.07	0.06	7.7E-03		
**NAD^+/*prs3***	0.30	0.38	0.43	0.44	0.61	0.66	0.65	0.72	0.39	0.66	0.07	0.05	7.7E-04	9.7E-05	1.6E-04

p>0.05
0.05<p>0.01
0.01<p>0.001
p<0.001
