# Supplementary material for: Dual control of NAD+ synthesis by purine metabolites in yeast
Source: eLife. 2019 Mar 12;8:e43808. doi: 10.7554/eLife.43808 (PMC6430606; doi:10.7554/eLife.43808)
Supplement: Figure 3—figure supplement 2—source data 2. [file elife-43808-fig3-figsupp2-data2.pdf]

Figure 3_figure supplement 2 C-D

FY4 wild-type strain grown in SD medium containing either 100 µM or 7.34 mM (concentration commonly supplied in SD medium) of inorganic phosphate (Pi)

Peak area

Metabolite / [Pi] (µM)	Sample 1	Sample 2	Sample 3	Sample 4	Sample 5	Sample 6	Mean	SD	Unpaired t-Test 7340 vs 100 µM Pi
ATP / 100	99.9	90.4	82.9	89.9	80.2	99.5	90.5	8.2	1.7E-05
ATP / 7340	110.4	131.4	127.5	131.2	128.3	128.4	126.2	7.9	

Metabolite / [Pi] (µM)	Sample 1	Sample 2	Sample 3	Sample 4	Sample 5	Sample 6	Mean	SD	Unpaired t-Test 7340 vs 100 µM Pi
NAD+ / 100	4.1	4.4	4.5	4.6	4.4	5.0	4.5	0.3	9.5E-06
NAD+ / 7340	6.5	8.2	7.8	7.3	7.5	7.1	7.4	0.6	

Relative peak area (mean peak area from cells grown in the presence 7.34 mM Pi was set at 1 and used to calculate the relative peak areas)

Metabolite / [Pi] (µM)	Sample 1	Sample 2	Sample 3	Sample 4	Sample 5	Sample 6	Mean	SD	Unpaired t-Test 7340 vs 100 µM Pi
ATP / 100	0.8	0.7	0.7	0.7	0.6	0.8	0.7	0.1	1.7E-05
ATP / 7340	0.9	1.0	1.0	1.0	1.0	1.0	1.0	0.1	

Metabolite / [Pi] (µM)	Sample 1	Sample 2	Sample 3	Sample 4	Sample 5	Sample 6	Mean	SD	Unpaired t-Test 7340 vs 100 µM Pi
NAD+ / 100	0.6	0.6	0.6	0.6	0.6	0.7	0.6	0.0	9.5E-06
NAD+ / 7340	0.9	1.1	1.1	1.0	1.0	1.0	1.0	0.1	

p>0.05
0.05<p>0.01
0.01<p>0.001
p<0.001
