# Supplementary material for: Dual control of NAD+ synthesis by purine metabolites in yeast
Source: eLife. 2019 Mar 12;8:e43808. doi: 10.7554/eLife.43808 (PMC6430606; doi:10.7554/eLife.43808)
Supplement: Figure 3—figure supplement 4—source data 1. [file elife-43808-fig3-figsupp4-data1.pdf]

Figure 3_figure supplement 4

FY4 wild-type strain grown in SGE(Glycerol Ethanol)casaWU ± Adenine medium

Peak area

																Mean	Mean	SD	SD	Unpaired t-Test
**Metabolite**	- Ade	- Ade	- Ade	- Ade	- Ade	- Ade	- Ade	+ Ade	+ Ade	+ Ade	+ Ade	+ Ade	+ Ade	+ Ade	+ Ade	- Ade	+ Ade	- Ade	+ Ade	**- Ade vs + Ade**
**ATP**	155.0	161.0	165.6	163.8	163.2	158.0	163.6	197.0	193.8	197.6	189.5	220.2	193.0	208.3	211.2	161.5	201.3	3.7	10.7	4.3E-06
**NAD+**	4.7	5.1	4.2	4.4	5.0	5.1	5.4	5.5	6.1	5.2	5.5	6.2	6.2	6.2	6.4	4.8	5.9	0.4	0.4	3.3E-04
**ADP**	36.9	34.8	40.7	40.8	38.8	42.1	43.3	45.7	44.5	44.6	46.1	50.2	46.4	46.0	50.8	39.6	46.8	3.0	2.4	
**AMP**	10.4	8.9	9.4	8.9	8.7	9.7	10.3	12.5	12.8	9.5	10.7	10.9	9.7	9.9	11.4	9.5	10.9	0.7	1.2	

Relative peak area **(mean peak area from cells grown in the presence of adenine was set at 1 and used to calculate the relative peak areas)**

																Mean	Mean	SD	SD	Unpaired t-Test
**Metabolite**	- Ade	- Ade	- Ade	- Ade	- Ade	- Ade	- Ade	+ Ade	+ Ade	+ Ade	+ Ade	+ Ade	+ Ade	+ Ade	+ Ade	- Ade	+ Ade	- Ade	+ Ade	**- Ade vs + Ade**
**ATP**	0.8	0.8	0.8	0.8	0.8	0.8	0.8	1.0	1.0	1.0	0.9	1.1	1.0	1.0	1.0	0.8	1.0	0.0	0.1	4.3E-06
**NAD+**	0.8	0.9	0.7	0.7	0.8	0.9	0.9	0.9	1.0	0.9	0.9	1.0	1.0	1.0	1.1	0.8	1.0	0.1	0.1	3.3E-04
**ADP**	0.8	0.7	0.9	0.9	0.8	0.9	0.9	1.0	1.0	1.0	1.0	1.1	1.0	1.0	1.1	0.8	1.0	0.1	0.1	
**AMP**	1.0	0.8	0.9	0.8	0.8	0.9	0.9	1.1	1.2	0.9	1.0	1.0	0.9	0.9	1.0	0.9	1.0	0.1	0.1	

Content

(nmol)

																Mean	Mean	SD	SD	Unpaired t-Test
**Metabolite**	- Ade	- Ade	- Ade	- Ade	- Ade	- Ade	- Ade	+ Ade	+ Ade	+ Ade	+ Ade	+ Ade	+ Ade	+ Ade	+ Ade	- Ade	+ Ade	- Ade	+ Ade	**- Ade vs + Ade**
**ATP**	8.07	8.39	8.63	8.53	8.50	8.23	8.52	10.26	10.09	10.29	9.87	11.47	10.05	10.85	11.00	8.4	10.5	0.2	0.6	4.3E-06
**ADP**	2.19	2.07	2.42	2.43	2.31	2.51	2.58	2.72	2.65	2.65	2.74	2.99	2.76	2.74	3.02	2.4	2.8	0.2	0.1	
**AMP**	0.54	0.46	0.48	0.46	0.45	0.50	0.53	0.65	0.66	0.49	0.55	0.56	0.50	0.51	0.59	0.5	0.6	0.0	0.1	
**AXP**	10.80	10.92	11.53	11.42	11.26	11.24	11.63	13.62	13.40	13.44	13.16	15.02	13.31	14.10	14.61	11.3	13.8	0.3	0.7	
**AEC**	0.85	0.86	0.85	0.85	0.86	0.84	0.84	0.85	0.85	0.86	0.85	0.86	0.86	0.87	0.86	0.9	0.9	0.0	0.0	7.5E-02

Relative content **(mean AXP content from cells grown in the presence of adenine was set at 1 and used to calculate relative content)**

																Mean	Mean	SD	SD	Unpaired t-Test
	- Ade	- Ade	- Ade	- Ade	- Ade	- Ade	- Ade	+ Ade	+ Ade	+ Ade	+ Ade	+ Ade	+ Ade	+ Ade	+ Ade	- Ade	+ Ade	- Ade	+ Ade	**- Ade vs + Ade**
**AXP**	0.78	0.79	0.83	0.83	0.81	0.81	0.84	0.98	0.97	0.97	0.95	1.09	0.96	1.02	1.06	0.8	1.0	0.0	0.0	2.0E-06

nmol/peak area unit (determined with pure compounds as described in the Material and Methods section

ATP	19.2
ADP	16.8
AMP	19.4

p>0.05
0.05<p>0.01
0.01<p>0.001
p<0.001
