# Supplementary material for: Dual control of NAD+ synthesis by purine metabolites in yeast
Source: eLife. 2019 Mar 12;8:e43808. doi: 10.7554/eLife.43808 (PMC6430606; doi:10.7554/eLife.43808)
Supplement: Figure 3—figure supplement 4—source data 2. [file elife-43808-fig3-figsupp4-data2.pdf]

Figure 3_figure supplement 4E

FY4 prototrophic cells grown in SGEcasaWU medium ± Adenine

													Mean	Mean	SD	SD	Unpaired t-test
	- Ade	- Ade	- Ade	- Ade	- Ade	- Ade	+ Ade	+ Ade	+ Ade	+ Ade	+ Ade	+ Ade	- Ade	+ Ade	- Ade	+ Ade	- Ade vs + Ade
**Median cell volume**	42	42.9	43.7	42.9	41.7	43	43	41	41	42.6	42.7	40.3	42.70	41.77	0.73	1.13	0.13

p>0.05
0.05<p>0.01
0.01<p>0.001
p<0.001
