# Supplementary material for: Dual control of NAD+ synthesis by purine metabolites in yeast
Source: eLife. 2019 Mar 12;8:e43808. doi: 10.7554/eLife.43808 (PMC6430606; doi:10.7554/eLife.43808)
Supplement: Figure 3—figure supplement 5—source data 1. [file elife-43808-fig3-figsupp5-data1.pdf]

# Figure 3_figure supplement 5

**Wild-type and mutant strains grown in SDcasaWU + Adenine medium**

## Peak area

| | | | | | | | Mean | SD | Unpaired t-Test |
|---|---|---|---|---|---|---|---|---|---|
| **Metabolite/Strain** | **+ Ade** | **+ Ade** | **+ Ade** | **+ Ade** | **+ Ade** | **+ Ade** | **+ Ade** | **+ Ade** | **WT vs mutant** |
| **ATP/WT** | 449.4 | 454.2 | 423.7 | 435.54 | 428.8 | 424 | 435.94 | 13.10 | |
| **ATP/*npt1*** | 435.7 | 454.4 | 441.4 | 447.8 | 432 | 458 | 444.88 | 10.33 | 2.2E-01 |
| **ATP/*kcs1*** | 949.7 | 922 | 873.8 | 959.4 | | | 926.23 | 38.37 | 5.1E-05 |
| **ATP/npt1 *kcs1*** | 1141.9 | 1221.8 | 1037.4 | 1085 | | | 1121.53 | 79.33 | 3.5E-04 |

| | | | | | | | Mean | SD | Unpaired t-Test |
|---|---|---|---|---|---|---|---|---|---|
| **Metabolite/Strain** | **+ Ade** | **+ Ade** | **+ Ade** | **+ Ade** | **+ Ade** | **+ Ade** | **+ Ade** | **+ Ade** | **WT vs mutant** |
| **$NAD^+$/WT** | 23.42 | 27.2 | 24.6 | 26.5 | 28.72 | 28.57 | 26.50 | 2.14 | |
| **$NAD^+$/*npt1*** | 6.4 | 6.45 | 8.3 | 7.7 | 7.87 | 7.82 | 7.42 | 0.80 | 4.6E-07 |
| **NAD+/*kcs1*** | 34.99 | 39.9 | 36.4 | 36.22 | | | 36.88 | 2.11 | 1.7E-04 |
| **NAD+/npt1 *kcs1*** | 5.95 | 4.72 | 5.35 | 5.38 | | | 5.35 | 0.50 | 5.9E-07 |

## Relative peak area **(mean peak area from wild-type cells was set at 1 and used to calculate the relative peak areas)**

| | | | | | | | Mean | SD | Unpaired t-Test |
|---|---|---|---|---|---|---|---|---|---|
| **Metabolite/Strain** | **+ Ade** | **+ Ade** | **+ Ade** | **+ Ade** | **+ Ade** | **+ Ade** | **+ Ade** | **+ Ade** | **WT vs mutant** |
| **ATP/WT** | 1.03 | 1.04 | 0.97 | 1.00 | 0.98 | 0.97 | 1.00 | 0.03 | |
| **ATP/*npt1*** | 1.00 | 1.04 | 1.01 | 1.03 | 0.99 | 1.05 | 1.02 | 0.02 | 2.2E-01 |
| **ATP/*kcs1*** | 2.18 | 2.11 | 2.00 | 2.20 | | | 2.12 | 0.09 | 5.1E-05 |
| **ATP/npt1 *kcs1*** | 2.62 | 2.80 | 2.38 | 2.49 | | | 2.57 | 0.18 | 3.5E-04 |

| | | | | | | | Mean | SD | Unpaired t-Test |
|---|---|---|---|---|---|---|---|---|---|
| **Metabolite/Strain** | **+ Ade** | **+ Ade** | **+ Ade** | **+ Ade** | **+ Ade** | **+ Ade** | **+ Ade** | **+ Ade** | **WT vs mutant** |
| **$NAD^+$/WT** | 0.88 | 1.03 | 0.93 | 1.00 | 1.08 | 1.08 | 1.00 | 0.08 | |
| **$NAD^+$/*npt1*** | 0.24 | 0.24 | 0.31 | 0.29 | 0.30 | 0.30 | 0.28 | 0.03 | 4.6E-07 |
| **NAD+/*kcs1*** | 1.32 | 1.51 | 1.37 | 1.37 | | | 1.39 | 0.08 | 1.7E-04 |
| **NAD+/npt1 *kcs1*** | 0.22 | 0.18 | 0.20 | 0.20 | | | 0.20 | 0.02 | 5.9E-07 |

| |
|---|
| p>0.05 |
| 0.05<p>0.01 |
| 0.01<p>0.001 |
| p<0.001 |
