# Supplementary material for: Dual control of NAD+ synthesis by purine metabolites in yeast
Source: eLife. 2019 Mar 12;8:e43808. doi: 10.7554/eLife.43808 (PMC6430606; doi:10.7554/eLife.43808)
Supplement: Figure 4—source data 1. [file elife-43808-fig4-data1.pdf]

Figure 4 B

Wild-type(BY4742) and *bas1 pho2* mutant cells were grown in SDcasaWU + Adenine and shifted at time 0 in SDcasaWU

Northern blot quantifications done with ImageQuant (GE Healthcare life sciences)

Strain	Time (min)	Experiment	*ACT1*	*ADE17*	*ADE17/ACT1*	Relative *ADE17/ACT1*	*BNA4*	*BNA4 / ACT1*	Relative *BNA4 / ACT1*	*BNA6*	*BNA6 / ACT1*	Relative *BNA6 / ACT1*	*PHO84*	*PHO84 / ACT1*	Relative *PHO84 / ACT1*
	0	1	2996	1840	0.6	**1.0**	278	0.09	**1.0**	56	0.02	**1.0**	8105	2.7	**1.0**
Wild-type	10	1	2711	22357	8.2	**13.4**	308	0.11	**1.2**	52	0.02	**1.0**	13349	4.9	**1.8**
	20	1	3416	38059	11.1	**18.1**	749	0.22	**2.4**	149	0.04	**2.3**	20087	5.9	**2.2**
	30	1	2973	24800	8.3	**13.6**	581	0.20	**2.1**	114	0.04	**2.1**	15960	5.4	**2.0**
	0	2	3244	2422	0.7	**1.0**	470	0.14	**1.0**	53	0.02	**1.0**	10205	3.1	**1.0**
Wild-type	10	2	2278	26882	11.8	**15.8**	427	0.19	**1.3**	30	0.01	**0.8**	15426	6.8	**2.2**
	20	2	3262	44414	13.6	**18.2**	923	0.28	**2.0**	100	0.03	**1.9**	23130	7.1	**2.3**
	30	2	3495	35220	10.1	**13.5**	987	0.28	**1.9**	130	0.04	**2.3**	22030	6.3	**2.0**
	0	3	4701	4926	1.0	**1.0**	62	0.01	**1.0**	588	0.13	**1.0**	29032	6.2	**1.0**
Wild-type	20	3	5166	93921	18.2	**17.4**	126	0.02	**1.8**	1295	0.25	**2.0**	65307	12.6	**2.0**
	30	3	4098	48594	11.9	**11.3**	102	0.02	**1.9**	1019	0.25	**2.0**	45940	11.2	**1.8**
	0	4	4235	5558	1.3	**1.0**	62	0.01	**1.0**	561	0.13	**1.0**	22268	5.3	**1.0**
Wild-type	20	4	5012	94001	18.8	**14.3**	132	0.03	**1.8**	1201	0.24	**1.8**	52918	10.6	**2.0**
	30	4	5058	71638	14.2	**10.8**	173	0.03	**2.3**	1384	0.27	**2.1**	57073	11.3	**2.1**
**Strain**	**Time (min)**	**Experiment**	***ACT1***	***ADE17***	***ADE17/ACT1***	**Relative *ADE17/ACT1***	***BNA4***	***BNA4 / ACT1***	**Relative *BNA4 / ACT1***	***BNA6***	***BNA6 / ACT1***	**Relative *BNA6 / ACT1***	***PHO84***	***PHO84 / ACT1***	**Relative *PHO84 / ACT1***
	0	1	3135	708	0.23	**1.0**	292	0.09	**1.0**	124	0.04	**1.0**	7822	2.5	**1.0**
bas1 pho2	10	1	2753	554	0.20	**0.9**	203	0.07	**0.8**	65	0.02	**0.6**	6201	2.3	**0.9**
	20	1	3228	938	0.29	**1.3**	392	0.12	**1.3**	75	0.02	**0.6**	7365	2.3	**0.9**
	30	1	2578	860	0.33	**1.5**	331	0.13	**1.4**	65	0.03	**0.6**	6686	2.6	**1.0**
	0	2	3644	1146	0.31	**1.0**	465	0.13	**1.0**	135	0.04	**1.0**	9828	2.7	**1.0**
bas1 pho2	10	2	3252	948	0.29	**0.9**	363	0.11	**0.9**	98	0.03	**0.8**	7391	2.3	**0.8**
	20	2	3696	1498	0.41	**1.3**	613	0.17	**1.3**	107	0.03	**0.8**	9018	2.4	**0.9**
	30	2	3005	1313	0.44	**1.4**	491	0.16	**1.3**	93	0.03	**0.8**	8642	2.9	**1.1**
	0	3	4016	1981	0.49	**1.0**	77	0.02	**1.0**	636	0.16	**1.0**	16488	4.1	**1.0**
bas1 pho2	20	3	3559	1404	0.39	**0.8**	63	0.02	**0.9**	430	0.12	**0.8**	11308	3.2	**0.8**
	30	3	3112	1255	0.40	**0.8**	73	0.02	**1.2**	339	0.11	**0.7**	11948	3.8	**0.9**
	0	4	3693	1886	0.51	**1.0**	75	0.02	**1.0**	551	0.15	**1.0**	8506	2.3	**1.0**
bas1 pho2	20	4	3592	1587	0.44	**0.9**	65	0.02	**0.9**	420	0.12	**0.8**	7083	2.0	**0.9**
	30	4	3184	1275	0.40	**0.8**	64	0.02	**1.0**	306	0.10	**0.6**	7154	2.2	**1.0**

Time (min)	Mean Relative ADE17/ACT1 Wild-type	SD Relative ADE17/ACT1 Wild-type	Mean Relative ADE17/ACT1 *bas1 pho2*	SD Relative ADE17/ACT1 *bas1 pho2*	Mean Relative BNA4 / ACT1 Wild-type	SD Relative BNA4 / ACT1 Wild-type	Mean Relative BNA4 / ACT1 *bas1 pho2*	SD Relative BNA4 / ACT1 *bas1 pho2*	Mean Relative BNA6 / ACT1 Wild-type	SD Relative BNA6 / ACT1 Wild-type	Mean Relative BNA6 / ACT1 *bas1 pho2*	SD Relative BNA6 / ACT1 *bas1 pho2*	Mean Relative PHO84 / ACT1 Wild-type	SD Relative PHO84 / ACT1 Wild-type	Mean Relative PHO84 / ACT1 *bas1 pho2*	SD Relative PHO84 / ACT1 *bas1 pho2*
0	**1.0**	0.0	**1.0**	0.0	**1.0**	0.0	**1.0**	0.0	**1.0**	0.0	**1.0**	0.0	**1.0**	0.0	**1.0**	0.0
10	**14.6**	1.7	**0.9**	0.0	**1.3**	0.0	**0.8**	0.1	**0.9**	0.2	**0.7**	0.2	**2.0**	0.2	**0.9**	0.0
20	**17.0**	1.9	**1.1**	0.3	**2.0**	0.3	**1.1**	0.2	**2.0**	0.2	**0.7**	0.1	**2.1**	0.1	**0.9**	0.1
30	**12.3**	1.5	**1.3**	0.4	**2.1**	0.2	**1.2**	0.2	**2.1**	0.1	**0.7**	0.1	**2.0**	0.1	**1.0**	0.1
