# Supplementary material for: Dual control of NAD+ synthesis by purine metabolites in yeast
Source: eLife. 2019 Mar 12;8:e43808. doi: 10.7554/eLife.43808 (PMC6430606; doi:10.7554/eLife.43808)
Supplement: Figure 4—figure supplement 1—source data 1. [file elife-43808-fig4-figsupp1-data1.pdf]

Figure 4 _ figure supplement 1 A

Wild-type cells (BY4742) grown in SDcasaWU and shifted at time 0 in SDcasaWU + Adenine

Time (min)	Phosphorimager Quantification *ACT1*	Phosphorimager Quantification *ADE17*	*ADE17 /ACT1*	**Relative *ADE17 /ACT1***	Phosphorimager Quantification *PHO84*	*PHO84 /ACT1*	**Relative *PHO84 /ACT1***	Phosphorimager Quantification *BNA6*	*BNA6 /ACT1*	**Relative *BNA6 /ACT1***	Phosphorimager Quantification *BNA4*	*BNA4 /ACT1*	**Relative *BNA4 /ACT1***
0	168	422	2.51	**1.00**	719	4.28	**1**	826	4.92	**1.0**	49292	293.40	**1.0**
10	167	130	0.78	**0.31**	1031	6.17	**1.4**	890	5.33	**1.1**	52930	316.95	**1.1**
20	200	22	0.11	**0.04**	579	2.90	**0.7**	884	4.42	**0.9**	48199	241.00	**0.8**
30	197	6	0.03	**0.01**	450	2.28	**0.5**	856	4.35	**0.9**	41019	208.22	**0.7**
45	212	14	0.07	**0.03**	381	1.80	**0.4**	637	3.00	**0.6**	23187	109.37	**0.4**
60	218	40	0.18	**0.07**	415	1.90	**0.4**	584	2.68	**0.5**	24066	110.39	**0.4**

Figure 4 _ figure supplement 1 B

Wild-type cells(BY4742) grown in SDcasaWU + Adenine and shifted at time 0 in SDcasaWU

Time (min)	Phosphorimager Quantification *ACT1*	Phosphorimager Quantification *ADE17*	*ADE17 /ACT1*	**Relative *ADE17 /ACT1***	Phosphorimager Quantification *PHO84*	*PHO84 /ACT1*	**Relative *PHO84 /ACT1***	Phosphorimager Quantification *BNA6*	*BNA6 /ACT1*	**Relative *BNA6 /ACT1***	Phosphorimager Quantification *BNA4*	*BNA4 /ACT1*	**Relative *BNA4 /ACT1***
0	181	71	0.39	**1.0**	218	1.20	**1.0**	2749	15.19	**1.0**	21668	119.7127072	**1.0**
10	225	848	3.77	**9.6**	576	2.56	**2.1**	5226	23.23	**1.5**	28455	126.4666667	**1.1**
20	233	1680	7.21	**18.4**	608	2.61	**2.2**	6442	27.65	**1.8**	51927	222.8626609	**1.9**
30	226	1588	7.03	**17.9**	613	2.71	**2.3**	8382	37.09	**2.4**	56172	248.5486726	**2.1**
45	213	829	3.89	**9.9**	497	2.33	**1.9**	9677	45.43	**3.0**	63589	298.5399061	**2.5**
60	208	699	3.36	**8.6**	516	2.48	**2.1**	10801	51.93	**3.4**	60921	292.8894231	**2.4**

Northern blot quantifications done with ImageQuant (GE Healthcare life sciences)
