# Supplementary material for: Dual control of NAD+ synthesis by purine metabolites in yeast
Source: eLife. 2019 Mar 12;8:e43808. doi: 10.7554/eLife.43808 (PMC6430606; doi:10.7554/eLife.43808)
Supplement: Figure 5—source data 1. [file elife-43808-fig5-data1.pdf]

Figure 5 A-F, M-N

WT, *ade16 ade17 and ade16 ade17 ade8 his1* knock-out strains grown in SDcasaWU + Adenine medium

Peak area

Metabolite/Strain	+ Ade	+ Ade	+ Ade	+ Ade	+ Ade	+ Ade	Mean + Ade	SD + Ade	Unpaired t-test WT or *ade16 ade17 ade8 his1* vs *ade16 ade17*	Unpaired t-test WTvs *ade16 ade17 ade8 his1*
ZMP/ WT	0.027	0.025	0.020	0.019	0.026	0.023	0.02	0.00	8.9E-09	
ZMP/ *ade16 ade17*	55.0	55.5	57.2	59.7	56.0	54.6	56.33	1.88		1.1E-05
ZMP/ *ade16 ade17 ade8 his1*	0.0	0.0	0.0	0.0	0.0	0.0	0.00	0.00	8.8E-09	

0 stands for non-detectable

Metabolite/Strain	+ Ade	+ Ade	+ Ade	+ Ade	+ Ade	+ Ade	Mean + Ade	SD + Ade	Unpaired t-test WT or *ade16 ade17 ade8 his1* vs *ade16 ade17*	Unpaired t-test WTvs *ade16 ade17 ade8 his1*
sZMP/ WT	0.0	0.0	0.0	0.0	0.0	0.0	0.00	0.00	2.3E-07	
SZMP/ *ade16 ade17*	16.0	17.8	16.8	17.8	16.0	15.2	16.60	1.06		Not Applicable
SZMP/ *ade16 ade17 ade8 his1*	0.0	0.0	0.0	0.0	0.0	0.0	0.00	0.00	2.3E-07	

0 stands for non-detectable

Metabolite/Strain	+ Ade	+ Ade	+ Ade	+ Ade	+ Ade	+ Ade	Mean + Ade	SD + Ade	Unpaired t-test WT or *ade16 ade17 ade8 his1* vs *ade16 ade17*	Unpaired t-test WTvs *ade16 ade17 ade8 his1*
ATP/ WT	174.2	170.2	157.0	172.2	173.3	183.0	171.7	8.4	1.5E-01	
ATP/ *ade16 ade17*	158.3	171.2	169.2	171.2	162.2	157.2	164.9	6.4		3.8E-01
ATP/ *ade16 ade17 ade8 his1*	165.7	148.5	175.5	168.3	168.3	174.5	166.8	9.8	7.0E-01	

Metabolite/Strain	+ Ade	+ Ade	+ Ade	+ Ade	+ Ade	+ Ade	Mean + Ade	SD + Ade	Unpaired t-test WT or *ade16 ade17 ade8 his1* vs *ade16 ade17*	Unpaired t-test WTvs *ade16 ade17 ade8 his1*
Trp/ WT	15.8	14.7	14.0	15.1	14.9	15.8	15.06	0.69	7.0E-07	
Trp/ *ade16 ade17*	10.2	10.9	11.0	11.2	10.3	10.5	10.68	0.42		1.9E-01
Trp/ *ade16 ade17 ade8 his1*	15.4	14.7	15.6	15.6	15.8	16.4	15.58	0.56	2.6E-08	

Metabolite/Strain	+ Ade	+ Ade	+ Ade	+ Ade	+ Ade	+ Ade	Mean + Ade	SD + Ade	Unpaired t-test WT or *ade16 ade17 ade8 his1* vs *ade16 ade17*	Unpaired t-test WTvs *ade16 ade17 ade8 his1*
Kynurenine/ WT	0.23	0.20	0.17	0.24	0.17	0.20	0.20	0.03	1.5E-12	
Kynurenine/ *ade16 ade17*	0.88	0.86	0.90	0.92	0.88	0.85	0.88	0.03		5.1E-01
Kynurenine/ *ade16 ade17 ade8 his1*	0.21	0.17	0.22	0.21	0.17	0.16	0.19	0.02	5.6E-13	

Metabolite/Strain	+ Ade	+ Ade	+ Ade	+ Ade	+ Ade	+ Ade	Mean + Ade	SD + Ade	Unpaired t-test WT or *ade16 ade17 ade8 his1* vs *ade16 ade17*	Unpaired t-test WTvs *ade16 ade17 ade8 his1*
3-OH-Kynurenine/ WT	0.070	0.063	0.053	0.034	0.040	0.060	0.05	0.01	7.0E-08	
3-OH-Kynurenine/ *ade16 ade17*	0.542	0.454	0.520	0.477	0.525	0.462	0.50	0.04		1.1E-02
3-OH-Kynurenine/ *ade16 ade17 ade8 his1*	0.032	0.030	0.036	0.032	0.034	0.026	0.03	0.00	5.7E-07	

Metabolite/Strain	+ Ade	+ Ade	+ Ade	+ Ade	+ Ade	+ Ade	Mean + Ade	SD + Ade	Unpaired t-test WT or *ade16 ade17 ade8 his1* vs *ade16 ade17*	Unpaired t-test WTvs *ade16 ade17 ade8 his1*
NAD^+/ WT	8.8	10.6	9.9	10.8	9.8	10.6	10.08	0.76	7.9E-01	
NAD^+/ *ade16 ade17*	9.4	9.9	10.3	10.4	9.1	10.7	9.97	0.62		8.6E-01
NAD^+/ *ade16 ade17 ade8 his1*	9.1	10.1	9.9	10.6	10.2	11.0	10.15	0.65	6.3E-01	

Metabolite/Strain	+ Ade	+ Ade	+ Ade	+ Ade	+ Ade	+ Ade	Mean + Ade	SD + Ade	Unpaired t-test WT or *ade16 ade17 ade8 his1* vs *ade16 ade17*	Unpaired t-test WTvs *ade16 ade17 ade8 his1*
Nicotinic acid/ WT	1.3	1.2	1.2	1.2	1.2	1.3	1.21	0.07	2.1E-01	
Nicotinic acid/ *ade16 ade17*	1.3	1.3	1.3	1.2	1.2	1.2	1.26	0.05		4.8E-01
Nicotinic acid/ *ade16 ade17 ade8 his1*	1.4	1.2	1.3	1.2	1.3	1.2	1.24	0.07	6.0E-01	

p>0.05
0.05<p>0.01
0.01<p>0.001
p<0.001
