# Supplementary material for: Dual control of NAD+ synthesis by purine metabolites in yeast
Source: eLife. 2019 Mar 12;8:e43808. doi: 10.7554/eLife.43808 (PMC6430606; doi:10.7554/eLife.43808)
Supplement: Figure 5—source data 2. [file elife-43808-fig5-data2.pdf]

Figure 5 G-L

ade16 ade17 ade8 his1 **quadruple knock-out strain grown in SDcawU + Adenine ± external AICAR (24 h)**

Peak area

Metabolite	AICAR No	AICAR No	AICAR 0.25 mM	AICAR 0.25 mM	AICAR 0.75 mM	AICAR 0.75 mM	AICAR 1 mM	AICAR 1 mM	AICAR 2 mM	AICAR 2 mM
**ZMP**	ND	ND	14.1	13.6	37.9	39.2	53.1	54.8	96	94.8
**SZMP**	ND	ND	5.27	5.19	14.2	16.2	20.1	18.9	27.65	27.97
**Kynurenine**	0.267	0.27	0.425	0.404	0.572	0.592	0.689	0.68	1.013	1.01
**3-OH-Kynurenine**	0.033	0.015	0.077	0.073	0.131	0.128	0.152	0.14	0.293	0.268
**3-OH-Anthranilate**	0.015	0.008	0.018	0.015	0.037	0.033	0.055	0.053	0.097	0.075
**Tryptophan**	36.8	35.5	29.6	29	26.1	26.7	26.3	26.1	23.9	25.1

ND: non-detectable
