# Supplementary material for: Dual control of NAD+ synthesis by purine metabolites in yeast
Source: eLife. 2019 Mar 12;8:e43808. doi: 10.7554/eLife.43808 (PMC6430606; doi:10.7554/eLife.43808)
Supplement: Figure 5—source data 3. [file elife-43808-fig5-data3.pdf]

Figure 5 O-Q

Wild-type and *npt1* knock-out strain grown in SDcasaWU ± Adenine medium

Peak area

Metabolite / strain	- Ade	- Ade	- Ade	- Ade	- Ade	- Ade	+ Ade	+ Ade	+ Ade	+ Ade	+ Ade	+ Ade	Mean - Ade	Mean + Ade	SD - Ade	SD + Ade	Unpaired t-test - Ade vs + Ade
NAD^+ / WT	19.4	17.3	21	20.1	20.9	21.3	22.8	21.9	25.4	24	24.6	26.2	20	24.15	1.493988	1.6046807	9.4E-04
NAD^+ / npt1	4.5	5.3	5.3	5.7	5.7	5.9	3.7	3.6	4.2	4.5	4.6	4.2	5.4	4.1333333	0.501996	0.4082483	8.2E-04

Metabolite / strain	- Ade	- Ade	- Ade	- Ade	- Ade	- Ade	+ Ade	+ Ade	+ Ade	+ Ade	+ Ade	+ Ade	Mean - Ade	Mean + Ade	SD - Ade	SD + Ade	Unpaired t-test - Ade vs + Ade
ZMP / WT	0.8	1.49	1.5	1.16	1.67	1.57	0.052	0.08	0.068	0.077	0.065	0.065	1.365	0.0678333	0.3256225	0.0099883	1.9E-04
ZMP / npt1	2.04	2.1	1.2	2.34	2.23	1.93	0.034	0.025	0.022	0.054	0.044	0.036	1.9733333	0.0358333	0.4051502	0.0119066	7.9E-05

Metabolite / strain	- Ade	- Ade	- Ade	- Ade	- Ade	- Ade	+ Ade	+ Ade	+ Ade	+ Ade	+ Ade	+ Ade	Mean - Ade	Mean + Ade	SD - Ade	SD + Ade	Unpaired t-test - Ade vs + Ade
SZMP / WT	2.5	4.36	4.26	3.61	3.83	4.21	0	0	0	0	0	0	3.795	0	0.6957226	0	4.2E-05
SZMP / npt1	5.2	4.65	2.42	4.1	4.52	4.52	0	0	0	0	0	0	4.235	0	0.9569065	0	1.2E-04

0 stand for non detectable

p>0.05
0.05<p>0.01
0.01<p>0.001
p<0.001
