# Supplementary material for: Dual control of NAD+ synthesis by purine metabolites in yeast
Source: eLife. 2019 Mar 12;8:e43808. doi: 10.7554/eLife.43808 (PMC6430606; doi:10.7554/eLife.43808)
Supplement: Figure 5—figure supplement 1—source data 1. [file elife-43808-fig5-figsupp1-data1.pdf]

Figure 5 _ figure supplement 1

Wild-type (WT) and *ade16 ade17* mutant strains grown in SDcasaWU medium -Nicotinic acid + Adenine

Peak area

Metabolite	WT	WT	WT	WT	WT	WT	WT	*ade16 ade17*	*ade16 ade17*	*ade16 ade17*	*ade16 ade17*	*ade16 ade17*	*ade16 ade17*	*ade16 ade17*	Mean WT	Mean *ade16 ade17*	SD WT	SD *ade16 ade17*	Unpaired t-test *ade16 ade17* vs WT
ATP	199.9	192.7	197.6	193.3	208	197	201.5	207.1	191.5	188.5	182	201	190	189.5	198.6	192.8	5.2	8.4	0.16
NAD^+	3.1	3.27	3.5	3.6	3.4	3.48	3.9	4.3	5.04	4.2	3.8	4.56	4.63	4.6	3.5	4.4	0.3	0.4	2.2E-04
ZMP	0.04	0.029	0.031	0.023	0.036	0.034	0.026	41.6	41.1	38.9	40.1	41.5	41.4	39.54	0.031	40.6	0.006	1.1	6.89E-11

Relative peak area (mean peak area from wild-type cells grown was set at 1 and used to calculate the relative peak areas)

Metabolite	WT	WT	WT	WT	WT	WT	WT	*ade16 ade17*	*ade16 ade17*	*ade16 ade17*	*ade16 ade17*	*ade16 ade17*	*ade16 ade17*	*ade16 ade17*	Mean WT	Mean *ade16 ade17*	SD WT	SD *ade16 ade17*	Unpaired t-test *ade16 ade17* vs WT
ATP	1.01	0.97	1.00	0.97	1.05	0.99	1.01	1.04	0.96	0.95	0.92	1.01	0.96	0.95	1.0	1.0	0.026402	0.04249755	0.16
NAD^+	0.89	0.94	1.01	1.04	0.98	1.00	1.13	1.24	1.45	1.21	1.10	1.32	1.34	1.33	1.0	1.3	0.0730611	0.113188928	2.2E-04
ZMP	1.28	0.93	0.99	0.74	1.15	1.09	0.83	1330	1314	1243	1282	1326	1323	1264	1.0	1297	0.1879371	34.43334157	6.89E-11

p>0.05
0.05<p>0.01
0.01<p>0.001
p<0.001
