# Supplementary material for: Dual control of NAD+ synthesis by purine metabolites in yeast
Source: eLife. 2019 Mar 12;8:e43808. doi: 10.7554/eLife.43808 (PMC6430606; doi:10.7554/eLife.43808)
Supplement: Figure 6—source data 1. [file elife-43808-fig6-data1.pdf]

Figure 6 A-B

WT and *kcs1* knock-out strains grown in SDcasaWU ± Adenine medium

Peak area

									Mean	Mean	SD	SD	Unpaired t-test
Metabolite/Strain	- Ade	- Ade	- Ade	- Ade	+ Ade	+ Ade	+ Ade	+ Ade	- Ade	+ Ade	- Ade	+ Ade	- Ade vs + Ade
Nicotinic acid/WT	3.43	3.74	3.43	3.74	2.3	2.9	2.3	2.63	3.59	2.53	0.18	0.29	1.6E-03
Nicotinic acid/*kcs1*	1.67	2.04	1.91		1.66	2.06		1.83	1.87	1.85	0.19	0.20	8.9E-01
ATP/WT	353.2	359.11	366.4	381.5	417.96	452	416.4	415.6	365.05	425.49	12.22	17.70	2.0E-03
ATP/*kcs1*	801	858.9	818.9		949.7	922		959.4	826.27	943.70	29.64	19.41	7.1E-03

Relative peak area (mean peak area from cells grown in the presence of adenine was set at 1 and used to calculate the relative peak areas)

									Mean	Mean	SD	SD	Unpaired t-test
Metabolite/Strain	- Ade	- Ade	- Ade	- Ade	+ Ade	+ Ade	+ Ade	+ Ade	- Ade	+ Ade	- Ade	+ Ade	- Ade vs + Ade
Nicotinic acid/WT	1.35	1.48	1.35	1.48	0.91	1.15	0.91	1.04	1.42	1.00	0.07	0.11	1.6E-03
Nicotinic acid/*kcs1*	0.66	0.81	0.75		0.66	0.81		0.72	0.74	0.73	0.07	0.08	8.9E-01
ATP/WT	0.83	0.84	0.86	0.90	0.98	1.06	0.98	0.98	0.86	1.00	0.03	0.04	2.0E-03
ATP/*kcs1*	1.88	2.02	1.92		2.23	2.17		2.25	1.94	2.22	0.07	0.05	7.1E-03

Non-determinable for technical reasons
mostly due to co-elution
in some samples

$p>0.05$
$0.05<p>0.01$
$0.01<p>0.001$
$p<0.001$
