# Supplementary material for: Dual control of NAD+ synthesis by purine metabolites in yeast
Source: eLife. 2019 Mar 12;8:e43808. doi: 10.7554/eLife.43808 (PMC6430606; doi:10.7554/eLife.43808)
Supplement: Figure 6—source data 2. [file elife-43808-fig6-data2.pdf]

Figure 6 C-D

Wild-type and *npt1* knock-out strain grown in SDcasaWU ± Adenine

Peak area

													Mean	Mean	SD	SD	Unpaired t-test
**Metabolite / strain**	- Ade	- Ade	- Ade	- Ade	- Ade	- Ade	+ Ade	+ Ade	+ Ade	+ Ade	+ Ade	+ Ade	- Ade	+ Ade	- Ade	+ Ade	**- Ade vs + Ade**
**Nicotinic acid / WT**	3.68	3.71	3.6	3.4	3.67	3.41	3.05	3.02	2.85	3	2.82	3.04	3.58	2.96	0.14	0.10	9.6E-06
**Nicotinic acid / npt1**	7.01	6.5	6.5	6.52	6.47	6.59	5.75	7.2	6.07	5.93	5.89	6.4	6.60	6.21	0.21	0.53	1.4E-01

													Mean	Mean	SD	SD	Unpaired t-test
**Metabolite / strain**	- Ade	- Ade	- Ade	- Ade	- Ade	- Ade	+ Ade	+ Ade	+ Ade	+ Ade	+ Ade	+ Ade	- Ade	+ Ade	- Ade	+ Ade	**- Ade vs + Ade**
**ATP / WT**	352.6	348.2	356.4	338.4	350.9	354.3	389.1	392.2	399.3	386.4	413.5	404.7	350	398	6	10	8.9E-06
**ATP / npt1**	397.3	386.8	400.2	370.5	376.3	397.5	413	424.2	406.7	439.1	417	431.4	388	422	12	12	7.4E-04

Relative peak area **(mean peak area from cells grown in the presence of adenine was set at 1 and used to calculate the relative peak areas)**

													Mean	Mean	SD	SD	Unpaired t-test
**Metabolite / strain**	- Ade	- Ade	- Ade	- Ade	- Ade	- Ade	+ Ade	+ Ade	+ Ade	+ Ade	+ Ade	+ Ade	- Ade	+ Ade	- Ade	+ Ade	**- Ade vs + Ade**
**Nicotinic acid / WT**	1.24	1.25	1.21	1.15	1.24	1.15	1.03	1.02	0.96	1.01	0.95	1.03	1.21	1.00	0.05	0.03	9.6E-06
**Nicotinic acid / npt1**	2.37	2.19	2.19	2.20	2.18	2.22	1.94	2.43	2.05	2.00	1.99	2.16	2.23	2.09	0.07	0.18	1.4E-01

													Mean	Mean	SD	SD	Unpaired t-test
**Metabolite / strain**	- Ade	- Ade	- Ade	- Ade	- Ade	- Ade	+ Ade	+ Ade	+ Ade	+ Ade	+ Ade	+ Ade	- Ade	+ Ade	- Ade	+ Ade	**- Ade vs + Ade**
**ATP / WT**	0.89	0.88	0.90	0.85	0.88	0.89	0.98	0.99	1.00	0.97	1.04	1.02	0.88	1.00	0.02	0.03	8.9E-06
**ATP / npt1**	1.00	0.97	1.01	0.93	0.95	1.00	1.04	1.07	1.02	1.10	1.05	1.09	0.98	1.06	0.03	0.03	7.4E-04

p>0.05
0.05<p>0.01
0.01<p>0.001
p<0.001
