# Supplementary material for: Dual control of NAD+ synthesis by purine metabolites in yeast
Source: eLife. 2019 Mar 12;8:e43808. doi: 10.7554/eLife.43808 (PMC6430606; doi:10.7554/eLife.43808)
Supplement: Figure 6—figure supplement 1—source data 1. [file elife-43808-fig6-figsupp1-data1.pdf]

Figure 6_figure supplement 1

bna2 and bna6 **knock-out strain grown in SDcasaWU ± Adenine medium**

Peak area

													Mean	Mean	SD	SD	Unpaired t-test
**Metabolite/Strain**	**- Ade**	**- Ade**	**- Ade**	**- Ade**	**- Ade**	**- Ade**	**+ Ade**	**+ Ade**	**+ Ade**	**+ Ade**	**+ Ade**	**+ Ade**	**- Ade**	**+ Ade**	**- Ade**	**+ Ade**	**- Ade vs + Ade**
**ATP/ *bna2***	174	162	154	147	139	153	196	199	199	200	198	178	154.83	195.00	12.12	8.44	9.6E-05
**NAD$^+$/ *bna2***	6.68	7	5.97	6.1	6.5	6.5	8.1	8.3	8.2	9	9.5	8.1	6.46	8.53	0.38	0.58	5.7E-05

Relative peak area (mean peak area from cells grown in the presence of adenine was set at 1 and used to calculate the relative peak areas)

													Mean	Mean	SD	SD	Unpaired t-test
**Metabolite/Strain**	**- Ade**	**- Ade**	**- Ade**	**- Ade**	**- Ade**	**- Ade**	**+ Ade**	**+ Ade**	**+ Ade**	**+ Ade**	**+ Ade**	**+ Ade**	**- Ade**	**+ Ade**	**- Ade**	**+ Ade**	**- Ade vs + Ade**
**ATP/ *bna2***	0.89	0.83	0.79	0.75	0.71	0.78	1.01	1.02	1.02	1.03	1.02	0.91	0.79	1.00	0.06	0.04	9.6E-05
**NAD$^+$/ *bna2***	0.78	0.82	0.70	0.71	0.76	0.76	0.95	0.97	0.96	1.05	1.11	0.95	0.76	1.00	0.04	0.07	5.7E-05

Peak area

							Mean	Mean	SD	SD	Unpaired t-test
**Metabolite**	**- Ade**	**- Ade**	**- Ade**	**+ Ade**	**+ Ade**	**+ Ade**	**- Ade**	**+ Ade**	**- Ade**	**+ Ade**	**- Ade vs + Ade**
**ATP**	291	290	286	339	333	352	289.00	341.33	2.65	32.32	7.7E-03
**NAD$^+$**	10.9	10.7	11.8	14.3	13.9	15.7	11.13	14.63	0.59	2.30	9.1E-03

p>0.05
0.05<p>0.01
0.01<p>0.001
p<0.001

Relative peak area (mean peak area from cells grown in the presence of adenine was set at 1 and used to calculate the relative peak areas)

							Mean	Mean	SD	SD	Unpaired t-test
**Metabolite**	**- Ade**	**- Ade**	**- Ade**	**+ Ade**	**+ Ade**	**+ Ade**	**- Ade**	**+ Ade**	**- Ade**	**+ Ade**	**- Ade vs + Ade**
**ATP**	1.46	1.46	1.44	1.70	1.67	1.77	1.45	1.72	0.01	0.16	7.7E-03
**NAD$^+$**	1.31	1.29	1.42	1.72	1.67	1.89	1.34	1.76	0.07	0.28	9.1E-03
