# Supplementary material for: Dual control of NAD+ synthesis by purine metabolites in yeast
Source: eLife. 2019 Mar 12;8:e43808. doi: 10.7554/eLife.43808 (PMC6430606; doi:10.7554/eLife.43808)
Supplement: Figure 6—figure supplement 2—source data 1. [file elife-43808-fig6-figsupp2-data1.pdf]

Figure 6_figure supplement 2

WT and mutant strains grown in SDcasaWU-Nicotinic acid + Nicotinamide ± Adenine medium

Peak area

Metabolite/Strain	- Ade	- Ade	- Ade	- Ade	+ Ade	+ Ade	+ Ade	+ Ade	Mean - Ade	Mean + Ade	SD - Ade	SD + Ade	Unpaired t-test - Ade vs + Ade
Nicotinic acid/WT	4.38	4.9	3.94	4.95	2.15	2.03	2.47	2.03	4.54	2.17	0.48	0.21	7.2E-04
Nicotinic acid/npt1	6.2	7.8	7.15	7.05	6.18	6.24	6.95	6.5	7.05	6.47	0.66	0.35	1.8E-01
Nicotinic acid/kcs1	2.86	2.95	2.82		2.81	3	2.94		2.88	2.92	0.07	0.10	5.9E-01

Relative peak area **(mean peak area from cells grown in the presence of adenine was set at 1 and used to calculate the relative peak areas)**

Metabolite/Strain	- Ade	- Ade	- Ade	- Ade	+ Ade	+ Ade	+ Ade	+ Ade	Mean - Ade	Mean + Ade	SD - Ade	SD + Ade	Unpaired t-test - Ade vs + Ade
Nicotinic acid/WT	2.02	2.26	1.82	2.28	0.99	0.94	1.14	0.94	2.09	1.00	0.22	0.10	7.2E-04
Nicotinic acid/npt1	2.86	3.59	3.29	3.25	2.85	2.88	3.20	3.00	3.25	2.98	0.30	0.16	1.8E-01
Nicotinic acid/kcs1	1.32	1.36	1.30		1.29	1.38	1.35		1.33	1.34	0.03	0.04	5.9E-01

Non-determinable for technical reasons
mostly due to co-elution
in some samples

p>0.05
0.05<p>0.01
0.01<p>0.001
p<0.001
