# Supplementary material for: Dual control of NAD+ synthesis by purine metabolites in yeast
Source: eLife. 2019 Mar 12;8:e43808. doi: 10.7554/eLife.43808 (PMC6430606; doi:10.7554/eLife.43808)
Supplement: Figure 7—source data 1. [file elife-43808-fig7-data1.pdf]

Figure 7 A-B

WT strain transformed by an *URA3* plasmid overexpressing (OE) the indicated gene were grown in SDcasaW (- Adenine) medium

Peak area

Figure 7 A-B

							Mean	SD	Unpaired t-test
Metabolite/overexpressed gene	- Ade	- Ade	- Ade	- Ade	- Ade	- Ade	- Ade	- Ade	OE gene vs None
ATP/None	185	175.4	146.9	167	169	141	164.05	16.89	
ATP/*NPT1*	167.9	160	158	163	158	168	162.48	4.61	8.3E-01
ATP/*NMA1*	165.7	172.7	159.4	153	129		155.96	16.75	4.5E-01
ATP/*NMA2*	160.2	148.3	167.9	183	156	142	159.57	14.61	6.3E-01
ATP/*QNS1*	161.6	177	181.4	181	151	140	165.33	17.33	9.0E-01

							Mean	SD	Unpaired t-test
Metabolite/overexpressed gene	- Ade	- Ade	- Ade	- Ade	- Ade	- Ade	- Ade	- Ade	OE gene vs None
NAD^+/None	8.5	8.49	9.89	8.8	9.8	9.3	9.13	0.63	
NAD^+/*NPT1*	11.9	9.16	8.4	7.97	9.3	8.8	9.26	1.39	8.5E-01
NAD^+/*NMA1*	14.3	14.03	14.87	13.9	12.2	13.8	13.85	0.89	2.3E-06
NAD^+/*NMA2*	10.5	8.97	10.6	10.3	9.8	8.5	9.78	0.87	1.7E-01
NAD^+/*QNS1*	9.45	9.4	9.57	9.9	9.6	9.7	9.60	0.18	1.3E-01

Relative peak area

(mean peak area from cells transformed with the empty vector (none) was set at 1 and used to calculate the relative peak areas)

Figure 7 A-B

							Mean	SD	Unpaired t-test
Metabolite/overexpressed gene	- Ade	- Ade	- Ade	- Ade	- Ade	- Ade	- Ade	- Ade	OE gene vs None
ATP/None	1.13	1.07	0.90	1.02	1.03	0.86	1.00	0.10	
ATP/*NPT1*	1.02	0.98	0.96	0.99	0.96	1.02	0.99	0.03	8.3E-01
ATP/*NMA1*	1.01	1.05	0.97	0.93	0.79		0.95	0.10	4.5E-01
ATP/*NMA2*	0.98	0.90	1.02	1.12	0.95	0.87	0.97	0.09	6.3E-01
ATP/*QNS1*	0.99	1.08	1.11	1.10	0.92	0.85	1.01	0.11	9.0E-01

							Mean	SD	Unpaired t-test
Metabolite/overexpressed gene	- Ade	- Ade	- Ade	- Ade	- Ade	- Ade	- Ade	- Ade	OE gene vs None
NAD^+/None	0.93	0.93	1.08	0.96	1.07	1.02	1.00	0.07	
NAD^+/*NPT1*	1.30	1.00	0.92	0.87	1.02	0.96	1.01	0.15	8.5E-01
NAD^+/*NMA1*	1.57	1.54	1.63	1.52	1.34	1.51	1.52	0.10	2.3E-06
NAD^+/*NMA2*	1.15	0.98	1.16	1.13	1.07	0.93	1.07	0.09	1.7E-01
NAD^+/*QNS1*	1.04	1.03	1.05	1.08	1.05	1.06	1.05	0.02	1.3E-01

Non-determinable for technical reasons mostly due to co-elution in some samples

p>0.05
0.05<p>0.01
0.01<p>0.001
p<0.001
