# Supplementary material for: Dual control of NAD+ synthesis by purine metabolites in yeast
Source: eLife. 2019 Mar 12;8:e43808. doi: 10.7554/eLife.43808 (PMC6430606; doi:10.7554/eLife.43808)
Supplement: Figure 7—source data 2. [file elife-43808-fig7-data2.pdf]

Figure 7 C-D

WT, *nma1* and *nma2* knock-out strains grown in SDcasaWU medium ± Adenine

Peak area

Metabolite/Strain	- Ade	- Ade	- Ade	- Ade	- Ade	- Ade	+ Ade	+ Ade	+ Ade	+ Ade	+ Ade	+ Ade	Mean - Ade	Mean + Ade	SD - Ade	SD + Ade	Unpaired t-test - Ade vs + Ade	Unpaired t-test mutant vs WT - Ade	Unpaired t-test mutant vs WT + Ade
ATP/WT	355	334.5	329	332.9	344	341.6	386	410.5	368	379	374.6	378.2	339.50	382.72	9.42	14.83	2.5E-04		
ATP/*nma1*	354	339.5	359	367	349	361	369	380.2	410	397.5	400.9	387.5	354.92	390.85	9.73	14.93	9.2E-04	1.9E-02	3.7E-01
ATP/*nma2*	324	335.5	337	319	319	342		370	379	389	382.5	388.9	329.42	381.88	9.99	7.90	4.6E-06	1.0E-01	9.1E-01

Metabolite/Strain	- Ade	- Ade	- Ade	- Ade	- Ade	- Ade	+ Ade	+ Ade	+ Ade	+ Ade	+ Ade	+ Ade	Mean - Ade	Mean + Ade	SD - Ade	SD + Ade	Unpaired t-test - Ade vs + Ade	Unpaired t-test mutant vs WT - Ade	Unpaired t-test mutant vs WT + Ade
NAD^+/WT	17.6	20.9	21.5	20.6	22.2	21.4	22.6	24.6	22.9	22.8	24	22.8	20.70	23.28	1.61	0.82	9.2E-03		
NAD^+/*nma1*	11.05	13.8	13.4	14.7	13.7	14.8	13.1	14.6	15.6	14.8	14.5	14.3	13.58	14.48	1.36	0.81	2.0E-01	1.1E-05	4.1E-09
NAD^+/*nma2*	18.1	19.8	20	18.7	18.5	19.2		21	23.6	22.5	23.1	22.2	19.05	22.48	0.75	0.99	3.0E-04	5.7E-02	1.9E-01

Relative peak area (mean peak area from wild-type cells grown in the presence of adenine was set at 1 and used to calculate the relative peak areas)

Metabolite/Strain	- Ade	- Ade	- Ade	- Ade	- Ade	- Ade	+ Ade	+ Ade	+ Ade	+ Ade	+ Ade	+ Ade	Mean - Ade	Mean + Ade	SD - Ade	SD + Ade	Unpaired t-test - Ade vs + Ade	Unpaired t-test mutant vs WT - Ade	Unpaired t-test mutant vs WT + Ade
ATP/WT	0.9276	0.874	0.8596	0.8698	0.8988	0.8926	1.0086	1.0726	0.9615	0.9903	0.9788	0.9882	0.89	1.00	0.02	0.04	2.5E-04		
ATP/*nma1*	0.925	0.8871	0.938	0.9589	0.9119	0.9433	0.9642	0.9934	1.0713	1.0386	1.0475	1.0125	0.93	1.02	0.03	0.04	9.2E-04	1.9E-02	3.7E-01
ATP/*nma2*	0.8466	0.8766	0.8805	0.8335	0.8335	0.8936		0.9668	0.9903	1.0164	0.9994	1.0162	0.86	1.00	0.03	0.02	4.6E-06	1.0E-01	9.1E-01

Metabolite/Strain	- Ade	- Ade	- Ade	- Ade	- Ade	- Ade	+ Ade	+ Ade	+ Ade	+ Ade	+ Ade	+ Ade	Mean - Ade	Mean + Ade	SD - Ade	SD + Ade	Unpaired t-test - Ade vs + Ade	Unpaired t-test mutant vs WT - Ade	Unpaired t-test mutant vs WT + Ade
NAD^+/WT	0.7559	0.8976	0.9234	0.8848	0.9535	0.9191	0.9707	1.0565	0.9835	0.9792	1.0308	0.9792	0.89	1.00	0.07	0.04	9.2E-03		
NAD^+/*nma1*	0.4746	0.5927	0.5755	0.6314	0.5884	0.6356	0.5626	0.6271	0.67	0.6356	0.6228	0.6142	0.58	0.62	0.06	0.03	2.0E-01	1.1E-05	4.1E-09
NAD^+/*nma2*	0.7774	0.8504	0.859	0.8031	0.7946	0.8246		0.9019	1.0136	0.9664	0.9921	0.9535	0.82	0.97	0.03	0.04	3.0E-04	5.7E-02	1.9E-01

Non-determinable for technical reasons mostly due to co-elution in some samples

p>0.05
0.05<p>0.01
0.01<p>0.001
p<0.001
