# Supplementary material for: Dual control of NAD+ synthesis by purine metabolites in yeast
Source: eLife. 2019 Mar 12;8:e43808. doi: 10.7554/eLife.43808 (PMC6430606; doi:10.7554/eLife.43808)
Supplement: Figure 7—source data 3. [file elife-43808-fig7-data3.pdf]

Figure 7 E-F

WT strain overexpressing (OE) or not (vector) NMA1 gene and grown in SDcasaW ± Adenine medium

Peak area

Metabolite/OE gene	- Ade	- Ade	- Ade	- Ade	+ Ade	+ Ade	+ Ade	+ Ade	Mean - Ade	Mean + Ade	SD - Ade	SD + Ade	Unpaired t-test - Ade vs + Ade	Unpaired t-test mutant vs WT - Ade	Unpaired t-test mutant vs WT + Ade
ATP/No	196	181.1	196.2	202	222.9	218.7	207.5	217.7	193.83	216.70	8.93	6.53	7.4E-03		
ATP/*NMA1*	186	196.6	200.2	205	217.5	223.4	211.8	216.3	196.95	217.25	8.07	4.78	8.0E-03	6.2E-01	9.0E-01

Metabolite/OE gene	- Ade	- Ade	- Ade	- Ade	+ Ade	+ Ade	+ Ade	+ Ade	Mean - Ade	Mean + Ade	SD - Ade	SD + Ade	Unpaired t-test - Ade vs + Ade	Unpaired t-test mutant vs WT - Ade	Unpaired t-test mutant vs WT + Ade
NAD^+/No	9.16	9.8	10.8	10.4	11.8	11.2	12.8	12.4	10.04	12.05	0.72	0.70	7.0E-03		
NAD^+/*NMA1*	11.9	14.2	13.7	14.3	15	16.75	16.3	17.3	13.53	16.34	1.11	0.98	9.4E-03	3.1E-03	6.0E-04

Relative peak area **(mean peak area from wild-type cells transformed with the empty vector (No) and grown in the presence of adenine was set at 1 and used to calculate the relative peak areas)**

Metabolite/OE gene	- Ade	- Ade	- Ade	- Ade	+ Ade	+ Ade	+ Ade	+ Ade	Mean - Ade	Mean + Ade	SD - Ade	SD + Ade	Unpaired t-test - Ade vs + Ade	Unpaired t-test mutant vs WT - Ade	Unpaired t-test mutant vs WT + Ade
ATP/No	0.9045	0.8357	0.9054	0.9322	1.0286	1.0092	0.9575	1.0046	0.89	1.00	0.04	0.03	7.4E-03		
ATP/*NMA1*	0.8583	0.9072	0.9239	0.946	1.0037	1.0309	0.9774	0.9982	0.91	1.00	0.04	0.02	8.0E-03	6.2E-01	9.0E-01

Metabolite/OE gene	- Ade	- Ade	- Ade	- Ade	+ Ade	+ Ade	+ Ade	+ Ade	Mean - Ade	Mean + Ade	SD - Ade	SD + Ade	Unpaired t-test - Ade vs + Ade	Unpaired t-test mutant vs WT - Ade	Unpaired t-test mutant vs WT + Ade
NAD^+/No	0.7602	0.8133	0.8963	0.8631	0.9793	0.9295	1.0622	1.029	0.83	1.00	0.06	0.06	7.0E-03		
NAD^+/*NMA1*	0.9876	1.1784	1.1369	1.1867	1.2448	1.39	1.3527	1.4357	1.12	1.36	0.09	0.08	9.4E-03	3.1E-03	6.0E-04

p>0.05
0.05<p>0.01
0.01<p>0.001
p<0.001
