# Supplementary material for: Dual control of NAD+ synthesis by purine metabolites in yeast
Source: eLife. 2019 Mar 12;8:e43808. doi: 10.7554/eLife.43808 (PMC6430606; doi:10.7554/eLife.43808)
Supplement: Figure 7—figure supplement 1—source data 1. [file elife-43808-fig7-figsupp1-data1.pdf]

Figure 7_figure supplement 1

WT strain transformed by an *URA3* plasmid overexpressing (OE) the indicated gene were grown in SDcasaW (- Adenine) medium

Peak area

Figure 7_figure supplement 1

							Mean	SD	Unpaired t-test
**Metabolite/overexpressed gene**	**- Ade**	**- Ade**	**- Ade**	**- Ade**	**- Ade**	**- Ade**	**- Ade**	**- Ade**	**OE gene vs None**
**NaMN/None**	0.78	0.34	0.363	0.56		0.66	0.54	0.19	
**NaMN/*NPT1***	2.3	2.21	2.28	3.9	4.1	3	2.97	0.85	6.9E-04
**NaMN/*NMA1***	0.065	0.084	0.087	0.05	0.054	0.05	0.07	0.02	4.8E-03
**NaMN/*NMA2***	0.392	0.398	0.397	0.56	0.48	0.52	0.46	0.07	4.0E-01
**NaMN/*QNS1***	0.365	0.417	0.447	0.58	0.5	0.59	0.48	0.09	5.6E-01

							Mean	SD	Unpaired t-test
**Metabolite/overexpressed gene**	**- Ade**	**- Ade**	**- Ade**	**- Ade**	**- Ade**	**- Ade**	**- Ade**	**- Ade**	**OE gene vs None**
**NaAD$^+$/None**	7.24	7.22	7.58	5.8	7	6.2	6.84	0.69	
**NaAD$^+$/*NPT1***	7.8	5.2	4.8	5.04	5.12	4.72	5.45	1.17	3.6E-02
**NaAD$^+$/*NMA1***	12.84	11.56	11.6	10.6	9.4		11.20	1.28	5.3E-04
**NaAD$^+$/*NMA2***	7.06	5.2	7.6	6.06	5.8	4.2	5.99	1.23	1.8E-01
**NaAD$^+$/*QNS1***	6.2	6.56	6.2	5	4.4		5.67	0.92	5.1E-02

Relative peak area **(mean peak area from cells transformed with the empty vector (none) was set at 1 and used to calculate the relative peak areas)**

Figure 7_figure supplement 1

							Mean	SD	Unpaired t-test
**Metabolite/overexpressed gene**	**- Ade**	**- Ade**	**- Ade**	**- Ade**	**- Ade**	**- Ade**	**- Ade**	**- Ade**	**OE gene vs None**
**NaMN/None**	1.44	0.63	0.67	1.04		1.22	1.00	0.35	
**NaMN/*NPT1***	4.25	4.09	4.22	7.21	7.58	5.55	5.48	1.58	6.9E-04
**NaMN/*NMA1***	0.12	0.16	0.16	0.09	0.10	0.09	0.12	0.03	4.8E-03
**NaMN/*NMA2***	0.73	0.74	0.73	1.04	0.89	0.96	0.85	0.13	4.0E-01
**NaMN/*QNS1***	0.68	0.77	0.83	1.07	0.92	1.09	0.89	0.17	5.6E-01

							Mean	SD	Unpaired t-test
**Metabolite/overexpressed gene**	**- Ade**	**- Ade**	**- Ade**	**- Ade**	**- Ade**	**- Ade**	**- Ade**	**- Ade**	**OE gene vs None**
**NaAD$^+$/None**	1.06	1.06	1.11	0.85	1.02	0.91	1.00	0.10	
**NaAD$^+$/*NPT1***	1.14	0.76	0.70	0.74	0.75	0.69	0.80	0.17	3.6E-02
**NaAD$^+$/*NMA1***	1.88	1.69	1.70	1.55	1.37		1.64	0.19	5.3E-04
**NaAD$^+$/*NMA2***	1.03	0.76	1.11	0.89	0.85	0.61	0.88	0.18	1.8E-01
**NaAD$^+$/*QNS1***	0.91	0.96	0.91	0.73	0.64		0.83	0.14	5.1E-02

Non-determinable for technical reasons
mostly due to co-elution
in some samples

p>0.05
0.05<p>0.01
0.01<p>0.001
p<0.001
