# Supplementary material for: Dual control of NAD+ synthesis by purine metabolites in yeast
Source: eLife. 2019 Mar 12;8:e43808. doi: 10.7554/eLife.43808 (PMC6430606; doi:10.7554/eLife.43808)
Supplement: Figure 7—figure supplement 2—source data 1. [file elife-43808-fig7-figsupp2-data1.pdf]

Figure 7_figure supplement 2

WT strain overexpressing *NPT1* gene and grown in SC - U ± Adenine medium

Peak area

									Mean	Mean	SD	SD	Unpaired t-test
**Metabolite/OE gene**	**- Ade**	**- Ade**	**- Ade**	**- Ade**	**+ Ade**	**+ Ade**	**+ Ade**	**+ Ade**	**- Ade**	**+ Ade**	**- Ade**	**+ Ade**	**- Ade vs + Ade**
**ATP/*NPT1***	327	296	271	267	441	416	425	450	290.25	433.00	27.66	15.34	3.9E-04
**NaMN/NPT1**	2.03	1.67	2.06	2.27	1.06	0.722	0.78	0.982	2.01	0.89	0.25	0.16	5.7E-04
**NAD^+/*NPT1***	17.2	12.5	15.2	15.7	19.2	18.5	21.5	22.7	15.15	20.48	1.96	1.96	8.5E-03

Relative peak area **(mean peak area from cells grown in the presence of adenine was set at 1 and used to calculate the relative peak areas)**

									Mean	Mean	SD	SD	Unpaired t-test
**Metabolite/Strain**	**- Ade**	**- Ade**	**- Ade**	**- Ade**	**+ Ade**	**+ Ade**	**+ Ade**	**+ Ade**	**- Ade**	**+ Ade**	**- Ade**	**+ Ade**	**- Ade vs + Ade**
**ATP/*NPT1***	0.7552	0.6836	0.6259	0.6166	1.0185	0.9607	0.9815	1.0393	0.67	1.00	0.06	0.04	3.9E-04
**NaMN/NPT1**	2.2912	1.8849	2.3251	2.5621	1.1964	0.8149	0.8804	1.1084	2.27	1.00	0.28	0.18	5.7E-04
**NAD^+/*NPT1***	0.84	0.6105	0.7424	0.7668	0.9377	0.9035	1.0501	1.1087	0.74	1.00	0.10	0.10	8.5E-03

p>0.05
0.05<p>0.01
0.01<p>0.001
p<0.001
