# Supplementary material for: Dual control of NAD+ synthesis by purine metabolites in yeast
Source: eLife. 2019 Mar 12;8:e43808. doi: 10.7554/eLife.43808 (PMC6430606; doi:10.7554/eLife.43808)
Supplement: Figure 8—source data 1. [file elife-43808-fig8-data1.pdf]

Figure 8 A-D

FY4 prototrophic wild-type strain grown in SDcasaWU ± Adenine medium

Peak area

																Mean	SD	Mean	SD	Unpaired t-test
**Metabolite**	**- Ade**	**- Ade**	**- Ade**	**- Ade**	**- Ade**	**- Ade**	**- Ade**	**- Ade**	**+ Ade**	**+ Ade**	**+ Ade**	**+ Ade**	**+ Ade**	**+ Ade**	**+ Ade**	**- Ade**	**- Ade**	**+ Ade**	**+ Ade**	**- Ade vs + Ade**
**NaMN**	0.46	0.46	0.64	0.63	0.58	0.54	0.6	0.72	0.35	0.43	0.5	0.44	0.44	0.52	0.48	0.58	0.07	0.45	0.04	6.0E-03
**NaAD+**	13	16.6	20	11.6	18	17.8	18	14.8	22.2	21	25	21.2	23.4	24.4	24.2	16.23	2.32	23.06	1.36	1.1E-04
**NAD+**	9.8	10.8	13.3	11.7	13.6	13.3	13.9	11.9	16.1	13.5	16.5	14.6	15.5	16.5	15.5	12.29	1.24	15.46	0.80	4.0E-04
**ATP**	322	287	337	319	293	313	289	297	353	344	367	336	353	363	359	307.13	15.63	353.57	8.08	6.3E-05

Relative peak area **(mean peak area from cells grown in the presence of adenine was set at 1 and used to calculate the relative peak areas)**

																Mean	SD	Mean	SD	Unpaired t-test
**Metabolite**	**- Ade**	**- Ade**	**- Ade**	**- Ade**	**- Ade**	**- Ade**	**- Ade**	**- Ade**	**+ Ade**	**+ Ade**	**+ Ade**	**+ Ade**	**+ Ade**	**+ Ade**	**+ Ade**	**- Ade**	**- Ade**	**+ Ade**	**+ Ade**	**- Ade vs + Ade**
**NaMN**	1.02	1.02	1.42	1.40	1.28	1.20	1.33	1.59	0.78	0.95	1.11	0.97	0.97	1.15	1.06	1.28	0.20	1.00	0.12	6.0E-03
**NaAD+**	0.56	0.72	0.87	0.50	0.78	0.77	0.78	0.64	0.96	0.91	1.08	0.92	1.01	1.06	1.05	0.70	0.12	1.00	0.07	1.1E-04
**NAD+**	0.63	0.70	0.86	0.76	0.88	0.86	0.90	0.77	1.04	0.87	1.07	0.94	1.00	1.07	1.00	0.79	0.10	1.00	0.07	4.0E-04
**ATP**	0.91	0.81	0.95	0.90	0.83	0.89	0.82	0.84	1.00	0.97	1.04	0.95	1.00	1.03	1.02	0.87	0.05	1.00	0.03	6.3E-05

p>0.05
0.05<p>0.01
0.01<p>0.001
p<0.001
