# Supplementary material for: Dual control of NAD+ synthesis by purine metabolites in yeast
Source: eLife. 2019 Mar 12;8:e43808. doi: 10.7554/eLife.43808 (PMC6430606; doi:10.7554/eLife.43808)
Supplement: Figure 8—figure supplement 1—source data 1. [file elife-43808-fig8-figsupp1-data1.pdf]

Figure 8 _ figure supplement 1

bna2 knock-out strains grown in SDcasaWU medium ± Adenine

Peak area

Metabolite	- Ade	- Ade	- Ade	- Ade	- Ade	- Ade	- Ade	- Ade	- Ade	- Ade	- Ade	- Ade	- Ade	- Ade	+ Ade	+ Ade	+ Ade	+ Ade	+ Ade	+ Ade	+ Ade	+ Ade	+ Ade	+ Ade	+ Ade	+ Ade	+ Ade	+ Ade	Mean - Ade	Mean + Ade	SD - Ade	SD + Ade	Unpaired t-test - Ade vs + Ade
ATP	178	187	160	162	174	174	197	198	222	204	207	188	207	217	217	222	219	240	254	217	216	229	213	251	226	247	225	222	191	228	19.53	13.77	5.5E-06
Nicotinic Acid	4.12	4.22	4.22	4.42	4.32	4.42	4.37	4.37							4.12	3.92	3.57	4.22	3.98	4.20	3.82	3.72							4.31	3.94	0.11	0.23	2.6E-03
NaMN	0.35	0.47	0.49	0.39	0.39	0.41	0.47	0.44							0.23	0.32	0.28	0.29	0.28	0.32	0.31	0.34							0.43	0.30	0.05	0.03	4.2E-05
$NaAD^+$	8.36	8.46	6.87	6.97	7.07	7.36	6.87	7.66	6.58	7.27	7.45	6.84	6.75	7.53	10.00	9.85	6.87	8.46	7.66	9.95	8.81	6.97	7.71	8.31	9.87	8.40	8.31	8.83	7.29	8.57	0.57	1.06	7.3E-04
NAD^+	6.48	8.18	9.28	9.18	7.88	9.47	9.18	9.18	7.68	8.08	6.89	6.99	7.48	7.48	8.98	8.58	8.98	11.97	9.47	11.17	10.17	9.47	9.36	9.55	9.45	10.34	10.93	9.36	8.10	9.84	1.00	0.96	7.5E-05

Relative peak area

Metabolite	- Ade	- Ade	- Ade	- Ade	- Ade	- Ade	- Ade	- Ade	- Ade	- Ade	- Ade	- Ade	- Ade	- Ade	+ Ade	+ Ade	+ Ade	+ Ade	+ Ade	+ Ade	+ Ade	+ Ade	+ Ade	+ Ade	+ Ade	+ Ade	+ Ade	+ Ade	Mean - Ade	Mean + Ade	SD - Ade	SD + Ade	Unpaired t-test - Ade vs + Ade
ATP	0.78	0.82	0.70	0.71	0.76	0.76	0.86	0.87	0.97	0.89	0.91	0.82	0.90	0.95	0.95	0.97	0.96	1.05	1.11	0.95	0.94	1.00	0.93	1.10	0.99	1.08	0.99	0.97	0.84	1.00	0.09	0.06	5.5E-06
Nicotinic Acid	1.04	1.07	1.07	1.12	1.10	1.12	1.11	1.11							1.04	0.99	0.90	1.07	1.01	1.07	0.97	0.94							1.09	1.00	0.03	0.06	2.6E-03
NaMN	1.18	1.58	1.65	1.31	1.31	1.38	1.58	1.50							0.77	1.08	0.94	0.99	0.94	1.08	1.04	1.15							1.44	1.00	0.17	0.11	4.2E-05
$NaAD^+$	0.98	0.99	0.80	0.81	0.82	0.86	0.80	0.89	0.77	0.85	0.87	0.80	0.79	0.88	1.17	1.15	0.80	0.99	0.89	1.16	1.03	0.81	0.90	0.97	1.15	0.98	0.97	1.03	0.85	1.00	0.07	0.12	7.3E-04
NAD^+	0.66	0.83	0.94	0.93	0.80	0.96	0.93	0.93	0.78	0.82	0.70	0.71	0.76	0.76	0.91	0.87	0.91	1.22	0.96	1.13	1.03	0.96	0.95	0.97	0.96	1.05	1.11	0.95	0.82	1.00	0.10	0.10	7.5E-05

p>0.05
0.05<p>0.01
0.01<p>0.001
p<0.001
