# Supplementary material for: Dual control of NAD+ synthesis by purine metabolites in yeast
Source: eLife. 2019 Mar 12;8:e43808. doi: 10.7554/eLife.43808 (PMC6430606; doi:10.7554/eLife.43808)
Supplement: Transparent reporting form [file elife-43808-transrepform.docx]

***eLife’s* transparent reporting form**

We encourage authors to provide detailed information *within their submission* to facilitate the interpretation and replication of experiments. Authors can upload supporting documentation to indicate the use of appropriate reporting guidelines for health-related research (see [EQUATOR Network](http://www.equator-network.org/%20)), life science research (see the [BioSharing Information Resource](https://biosharing.org/)), or the [ARRIVE guidelines](http://www.plosbiology.org/article/info:doi/10.1371/journal.pbio.1000412) for reporting work involving animal research. Where applicable, authors should refer to any relevant reporting standards documents in this form.

If you have any questions, please consult our Journal Policies and/or contact us: [editorial@elifesciences.org](mailto:editorial@elifesciences.org).

**Sample-size estimation**

- You should state whether an appropriate sample size was computed when the study was being designed
- You should state the statistical method of sample size computation and any required assumptions
- If no explicit power analysis was used, you should describe how you decided what sample (replicate) size (number) to use

Please outline where this information can be found within the submission (e.g., sections or figure legends), or explain why this information doesn’t apply to your submission:

For metabolic analyses, we only performed biological replicates (independent cell cultures and metabolite extractions). Indeed, in our experience on our HPIC device, technical replicates are not relevant due to extremely high reproducibility between two independent injections and separations from the same biological sample.

The number of biological replicates was set up at eight in the initial experiment, but due to high significance and combination of redundant approaches, the number of replicates was reduced in further experiments (N ≥ 3).

In the dose-response experiment (Figure 5G-L) in which each point is “consolidated” by neighboring values, N was 2. Similarly, in the northern blot kinetic analyses (Figure 4_figure supplement 1), each time point is “consolidated” by neighboring values (N was 1) and by an independent approach (Figure 4C-D). Northern blot from figure 4 B was done on 2 (time point 10 min) to 4 independent RNA extractions (time points 0, 20 and 30 min).

**Replicates**

- You should report how often each experiment was performed
- You should include a definition of biological versus technical replication
- The data obtained should be provided and sufficient information should be provided to indicate the number of independent biological and/or technical replicates
- If you encountered any outliers, you should describe how these were handled
- Criteria for exclusion/inclusion of data should be clearly stated
- High-throughput sequence data should be uploaded before submission, with a private link for reviewers provided (these are available from both GEO and ArrayExpress)

Please outline where this information can be found within the submission (e.g., sections or figure legends), or explain why this information doesn’t apply to your submission:

The number N of independent biological replicates for each experiment is indicated in the corresponding figure and figure supplement legends.

We only performed biological replicates: independent cell cultures and sample treatment.

No outliers.

Exclusion of data upon metabolic analyses (gray cells in the raw data excel sheet uploaded) correspond to non-determinable values for technical reasons mostly due to chromatographic co-elution in some samples (mixed peaks, could not be quantified).

**Statistical reporting**

- Statistical analysis methods should be described and justified
- Raw data should be presented in figures whenever informative to do so (typically when N per group is less than 10)
- For each experiment, you should identify the statistical tests used, exact values of N, definitions of center, methods of multiple test correction, and dispersion and precision measures (e.g., mean, median, SD, SEM, confidence intervals; and, for the major substantive results, a measure of effect size (e.g., Pearson's r, Cohen's d)
- Report exact p-values wherever possible alongside the summary statistics and 95% confidence intervals. These should be reported for all key questions and not only when the p-value is less than 0.05.

Please outline where this information can be found within the submission (e.g., sections or figure legends), or explain why this information doesn’t apply to your submission:

Statistics were given as p-values determined by a Welch’s unpaired t-test assuming a bilateral distribution and unequal variances. Welch's t-test is more robust than Student's t-test and maintains type I error (rejection of the true null hypothesis) rates close to nominal for unequal variances.

Raw data are shown in Figures and Figure supplements since they are presented by scattered plots with mean and SD. N and exact p-values are indicated in the legend and the figure itself, respectively. In addition, all statistics can be found in the raw data excel sheet uploaded.

(For large datasets, or papers with a very large number of statistical tests, you may upload a single table file with tests, Ns, etc., with reference to sections in the manuscript.)

**Group allocation**

- Indicate how samples were allocated into experimental groups (in the case of clinical studies, please specify allocation to treatment method); if randomization was used, please also state if restricted randomization was applied
- Indicate if masking was used during group allocation, data collection and/or data analysis

Please outline where this information can be found within the submission (e.g., sections or figure legends), or explain why this information doesn’t apply to your submission:

Not applicable

**Additional data files (“source data”)**

- We encourage you to upload relevant additional data files, such as numerical data that are represented as a graph in a figure, or as a summary table
- Where provided, these should be in the most useful format, and they can be uploaded as “Source data” files linked to a main figure or table
- Include model definition files including the full list of parameters used
- Include code used for data analysis (e.g., R, MatLab)
- Avoid stating that data files are “available upon request”

Please indicate the figures or tables for which source data files have been provided:

All data have been provided in the raw data excel sheet uploaded.
